# Pickering emulsions stabilized by coloured organic pigment particles†
†Electronic supplementary information (ESI) available. See DOI: 10.1039/c6sc03085h


**DOI:** 10.1039/c6sc03085h

**Published:** 2016-09-19

**Authors:** Bernard P. Binks, Samuel O. Olusanya

**Affiliations:** a Department of Chemistry , University of Hull , Hull HU6 7RX , UK . Email: b.p.binks@hull.ac.uk

## Abstract

The possibility of stabilizing emulsions of water and non-polar alkane with pure, coloured organic pigment particles is explored. Seven pigment types each possessing a primary colour of the rainbow were selected. Their solubility in water or heptane was determined using a spectrophotometric method and their surface energies were derived from the contact angles of probe liquids on compressed disks of the particles. As expected, most of the pigments are relatively hydrophobic but pigment orange is quite hydrophilic. At equal volumes of oil and water, preferred emulsions were water-in-oil (w/o) for six pigment types and oil-in-water (o/w) for pigment orange. The emulsion type is in line with calculated contact angles of the particles at the oil–water interface being either side of 90°. Their stability to coalescence increases with particle concentration. Emulsions are shown to undergo limited coalescence from which the coverage of drop interfaces by particles has been determined. In a few cases, close-packed primary particles are visible around emulsion droplets. At constant particle concentration, the influence of the volume fraction of water (*φ*_w_) on emulsions was also studied. For the most hydrophilic pigment orange, emulsions are o/w at all *φ*_w_, whereas they are w/o for the most hydrophobic pigments (red, yellow, green and blue). For pigments of intermediate hydrophobicity however (indigo and violet), catastrophic phase inversion becomes possible with emulsions inverting from w/o to o/w upon increasing *φ*_w_. For the first time, we link the pigment surface energy to the propensity of emulsions to phase invert transitionally or catastrophically.

## Abstract 2

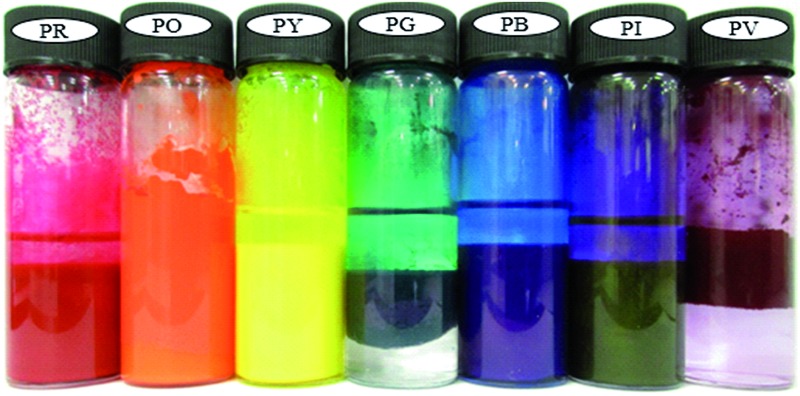
Pickering emulsions stabilised by coloured organic pigment particles.

## Introduction

Pigments are particulate materials that have colour imparting ability and they are largely insoluble in their medium of application which is due to the inherent intermolecular aggregation through hydrogen bonding as well as van der Waals forces.^1–3^ Pigments of different colour exist and the difference in colour is due to the variation in the colour absorbing ability of each pigment, related to differences in their chemical structure.^4^ Pigments can be classified basically into two types, organic pigments and inorganic pigments.^5^ Organic pigments are composed of chemical compounds containing carbon and hydrogen along with other elements like oxygen, sulphur or nitrogen^6^ while inorganic pigments are colourants that consist of mineral compounds like oxides or sulphides of one or more metals.^7^ Of the many ways in which light can interact with objects, the two most important in terms of their influence on colour are absorption and scattering. Absorption is the process by which radiant energy is used to raise molecules in the object to higher energy states. Scattering is the interaction by which light is re-directed as a result of multiple refractions and reflections. If only absorption is involved, the object will appear transparent. If scattering centers are present, the object will appear either translucent or opaque as light is reflected back to the observer. When pigments absorb light of a specific wavelength (corresponding to a colour), its complementary colour is what will be observed as the colour of the pigment, *i.e*. colour corresponding to the wavelength of transmitted light.^8^ Pigments are used extensively in many different products like ink,^9^ cosmetics,^10^ surface coatings,^11^ paint and in electronic devices for liquid crystal displays and electrostatically charged toners.^12,13^ In all of these applications, the pigment particles are used in conjunction with other materials including polymers, surfactants, other particles, solvents *etc.* and it is sometimes unclear if pigment particles remain in bulk, reside at an interface or partition between the two in the final end product.

The main aim of the present work is to investigate the behavior of coloured pigment particles at oil–water interfaces in the absence of other components. Can pigment particles act as the stabilizer of emulsions in addition to imparting colour? Although reports do exist describing emulsion stabilization by inorganic pigments and carbon, no study exists employing organic pigment particles of different colour despite their widespread use. Different types of carbon particles have been used as sole emulsifiers including lamp black,^14^ carbon black^15,16^ and carbon nanotubes.^17^ Emulsions stabilized by so-called amphiphilic carbon nanotubes were described by Wang and Hobbie.^18^ The formation of stable oil-in-water (o/w) emulsions was also shown possible with modified carbon nanotubes as emulsifier.^19^ Particle-stabilised or Pickering emulsions have also been prepared recently using uniform carbon microspheres^20^ and either single-wall^21^ or multi-wall carbon nanotubes.^22,23^ Similarly, white particles of titania when suitably chemically modified were shown to be effective emulsifiers of oil and water,^24,25^ as have brown microparticles of carbonyl iron^26^ or nanoparticles of iron oxide.^27^

For this paper, we selected seven types of organic pigment particle, each one being a primary colour of the rainbow (red, orange, yellow, green, blue, indigo and violet). We determined their surface energy using contact angle measurements and measured their (low) solubility in water and oil using spectrophotometry. We explore the possibility of stabilising emulsions of oil and heptane with such pigment particles alone. At fixed oil : water ratio of unity, the effect of particle concentration on emulsion type and stability is studied in detail including analysis of particle coverage around droplets and microscopy evidence of their arrangement. Catastrophic inversion by varying the oil : water ratio is demonstrated in some cases, and we correlate the propensity for phase inversion with the pigment surface energy.

## Experimental

### Materials

Seven pigments each possessing a primary colour of the rainbow were received from BASF, Germany. They were used without any further purification. Their names, abbreviations and other properties are given in Table 1. The pigments are crystalline in nature with an average primary particle diameter of a few hundred nanometres. The densities of the particles range from 1.32 to 2.14 g cm^–3^. Pigments PR and PO are mono-azo pigments, PY is a quinophthalone whilst PG and PB belong to the phthalocyanine class. PI is a dioxazine pigment and PV is a quinacridone pigment. The oil used for preparing emulsions was *n*-heptane (Sigma Aldrich, 99% pure). It was columned twice before use (using basic alumina from Merck) in order to remove polar impurities. Water was passed through an Elga reverse osmosis unit and then a Milli-Q reagent water system. Four other liquids used for the estimation of pigment surface energy were glycerol (Sigma, 99%), formamide (Sigma, >99%), α-bromonapthalene (Sigma, 97%) and *n*-hexadecane (Sigma, 99%).

**Table 1:** Abbreviation, name, structure, particle diameter and density of the pigments used

Abbreviation	Name	Chemical structure	Primary particle diameter/±0.05 μm	Particle density/g cm^–3^ at 20 °C
PR	Irgalite Red D 3707 (Pigment Red 23)	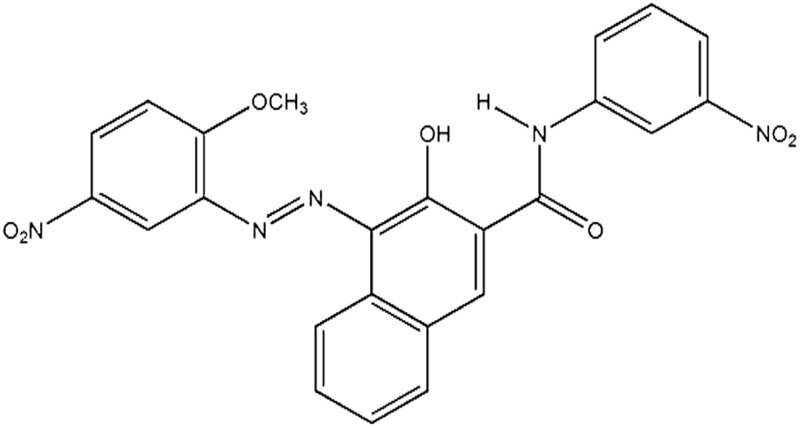	0.16	1.40
PO	Cromophtal K 2960 (Pigment Orange 64)	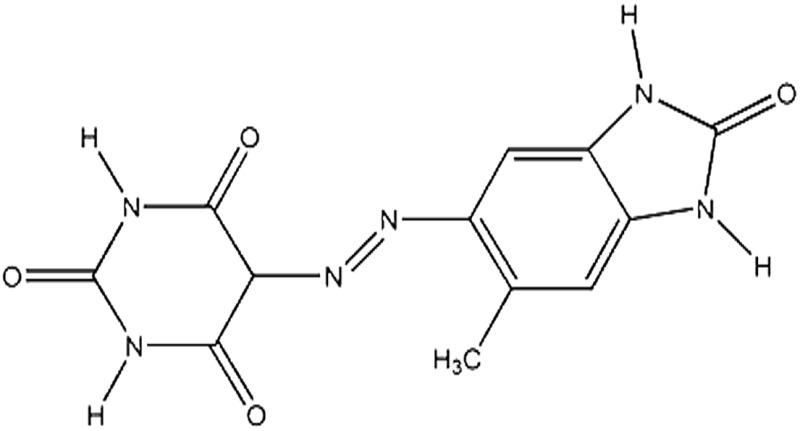	0.18	1.59
PY	Paliotol Yellow K 0961 (C.I.^*a*^ Pigment Yellow 138)	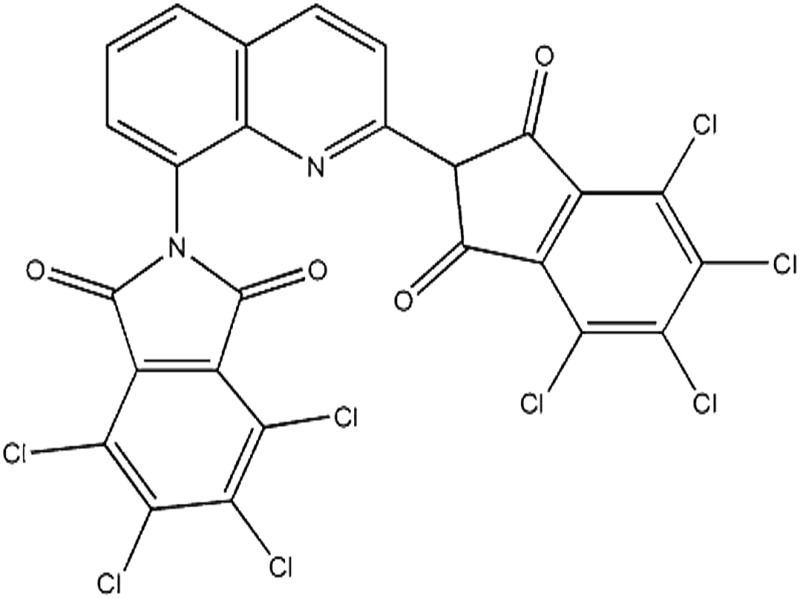	0.17	1.80
PG	Heliogen Grün K 8730 (C.I. Pigment Green 7)	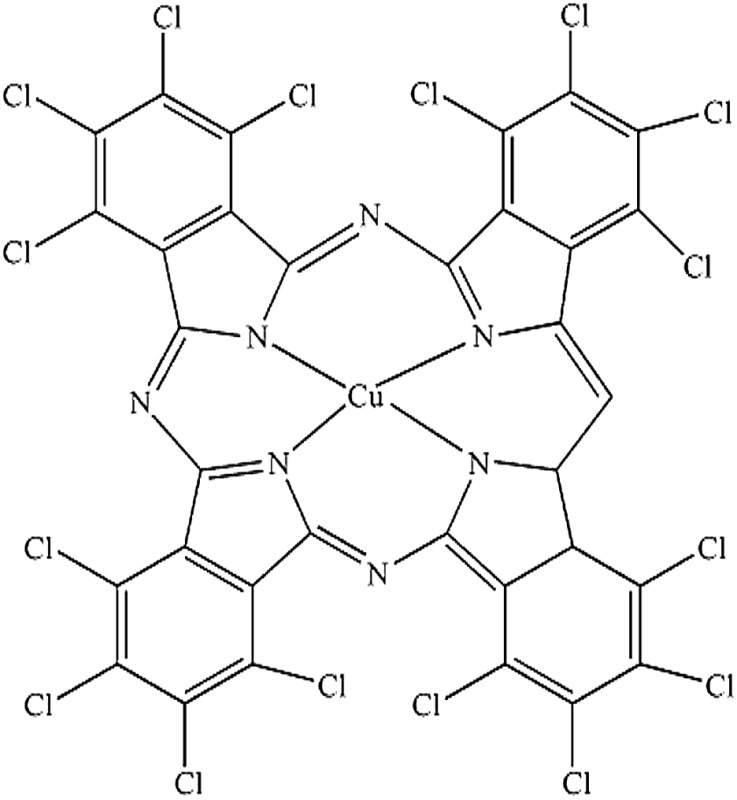	0.16	2.14
PB	Heliogen Blau K 6907 (C.I. pigment 15 : 1)	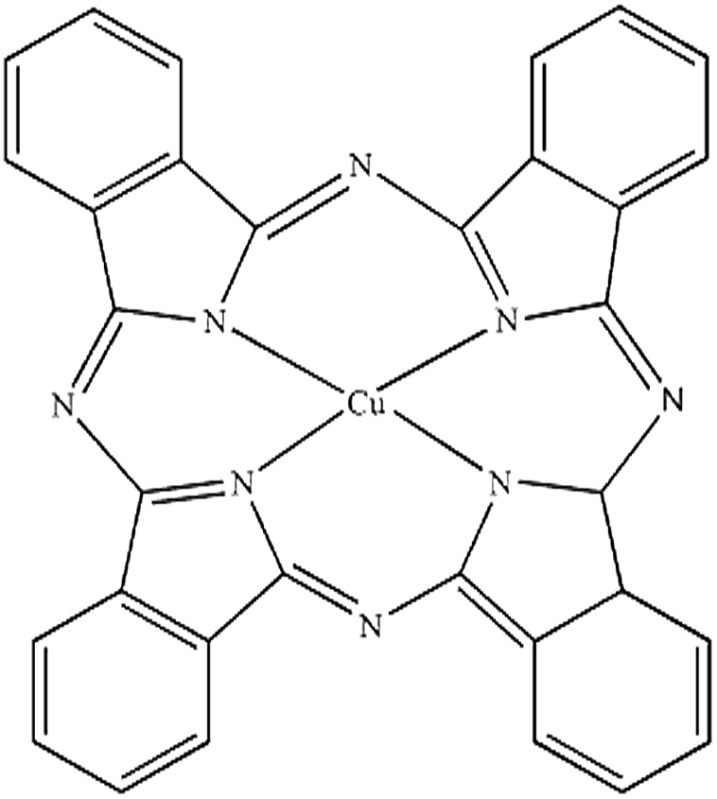	0.44	1.60
PI	Cromophtal Violet D 5700 (C.I. pigment violet 37)	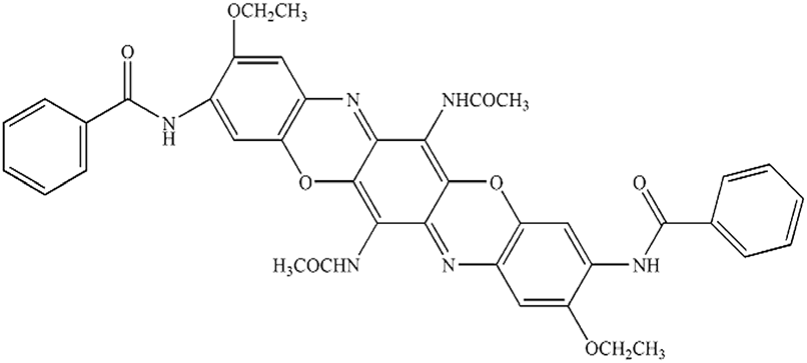	0.15	1.32
PV	Cinquasia Violet L 5120 (C.I. Pigment Violet 19)	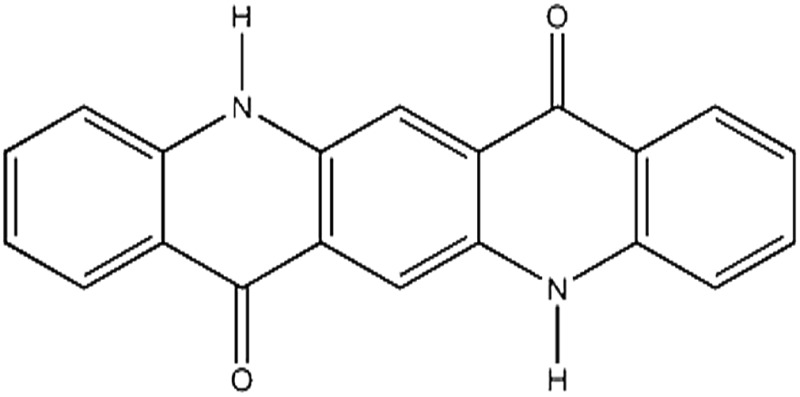	0.12	1.49

^*a*^C.I. = colour index.

### Methods

#### (i) Characterization of pigment particles by SEM and EDX

**Fig. 1:**
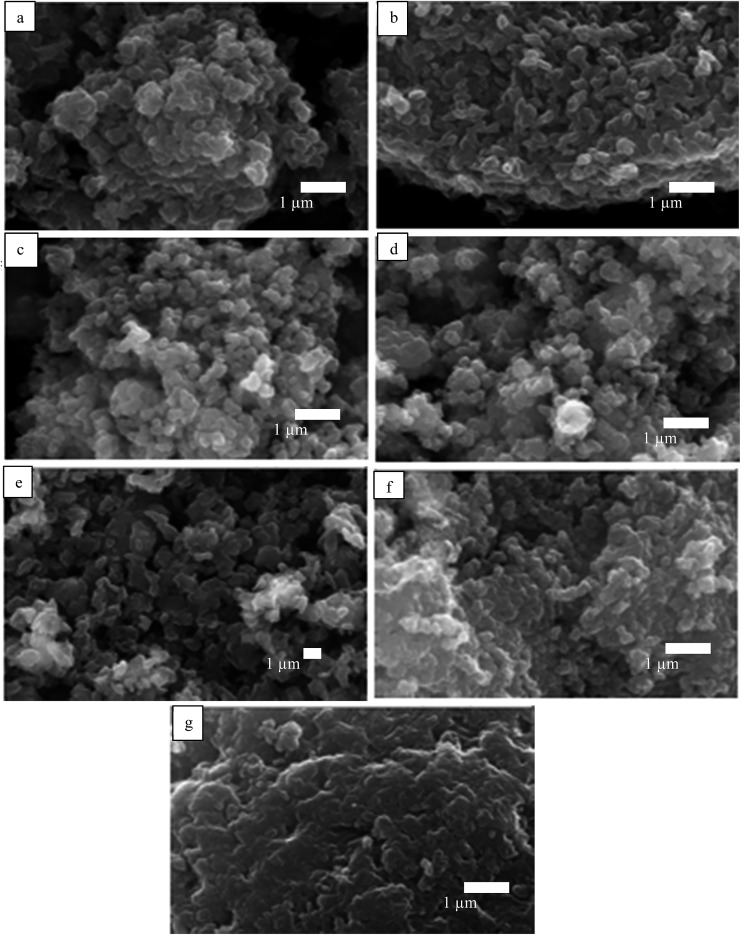
SEM images of powders of the seven pigments showing the primary particles: (a) PR, (b) PO, (c) PY, (d) PG, (e) PB, (f) PI, (g) PV.

The appearance of the primary particles in each pigment sample was probed by scanning electron microscopy (SEM). A thin layer (12 mm diameter) of ‘sticky’ carbon disc was applied to a standard 12 mm aluminum SEM stub. Then, a small quantity of pigment powder was placed on a clean sheet of paper and the ‘sticky’ stub was gently brought into contact with it. Excess loose material was removed using low pressure compressed air. The mounted samples were coated with a 10–15 nm layer of carbon using an Edwards vacuum evaporator. Thereafter, the samples were examined and imaged with a Zeiss EVO 60 SEM using a beam voltage of 20 kV and probe current of 100 pA. The SEM images are shown in Fig. 1. The average diameter of the primary particles was determined with ImageJ by random selection of 200 particles (Table 1); agglomerates exist ranging in size to 25 μm (Fig. S1†). All the pigments have primary particles with quasi-spherical shape, although those of PB and PV are more non-spherical. For energy-dispersive X-ray analysis (EDX), ≈200 mg of sample was compressed by applying a pressure of 10^9^ N m^–2^ using a hydraulic press in order to produce a 12 mm diameter pellet with a flat surface. The pellet was attached to a standard 12 mm Al stub using super-glue. No further preparation was necessary as the sample pellets were analyzed under variable pressure conditions in the SEM (about 30 Pa). EDX analyses were obtained under identical SEM conditions as above and the EDX spectra obtained are shown in Fig. S2.† The elemental compositions of the seven pigments are given in Table S1.†

#### (ii) Determination of pigment surface energy and measurement of oil–water contact angle

For particles which are irregular and polydisperse, difficulties exist in the direct measurement of the contact angle at a liquid surface. For this purpose, disks (diameter, 13 mm; thickness, 2 mm) were made from the pigment particles by compressing 400 mg of pigment powder in a steel die using a hydraulic press (Research and Industrial Instrument Co., UK) with a pressure of 10^9^ N m^–2^. Some disks (PY, PG, PB and PI) were supported on aluminium foil with their side taped with parafilm to provide mechanical support. The oil–water and liquid–air contact angles were measured using a Krüss DSA Mk 10 apparatus. For the surface energy estimation, the advancing contact angle of a drop of water or other liquids in air was measured by placing 10 μL on the surface of the pigment disk. For PO which dissolves slightly in water, we checked that the air–water surface tension was not lowered from its bare value. The oil–water contact angle was measured by placing the disk in a cubical quartz cell (dimension, 2 × 2 × 2 cm) and filling the cell with 3 cm^3^ of *n*-heptane. Thereafter, a drop of water was gently placed on the surface of the disk and the contact angle was measured through water. For certain disks, a drop of water was first placed on the surface of the disk in air and then the cell was gently filled with oil. Three different readings were taken and the average was used.^28^

#### (iii) Determination of pigment solubility by spectrophotometric method

The molecular solubility of the pigments was determined spectrophotometrically at room temperature in water or *n*-heptane as described by Vu-Duc *et al.* ^29^ Absorbance measurements were performed with a UV-Vis spectrophotometer (Perkin Elmer Lambda) using a quartz cuvette of path length 1 cm. In order to determine the wavelength corresponding to maximum absorption (*λ*_max_) of each pigment, 1 mg of each pigment was dispersed in 10 mL of water or *n*-heptane. The mixture was vigorously hand shaken for 30 s. The unfiltered diluted dispersions were scanned between 250 and 700 nm to determine *λ*_max_. The dispersions were then filtered with Whatman filter paper no. 1002–090 (pore size 8 μm) and centrifuged with a Biofuge Primo centrifuge operating at 5000 rpm for ten minutes thus separating the fine particles. The absorbance of the supernatant was then measured at *λ*_max_. The apparent extinction coefficients for the seven pigments were determined as follows. Dispersions with pigment concentration ranging from 0.03 to 0.14 mg mL^–1^ were prepared in batch. The absorbance was measured immediately after preparation. At least three absorbance readings were taken for each dispersion. A calibration curve (absorbance *vs.* concentration) was produced. The solubility of the pigment in the solvent was determined from the absorbance of the supernatant and the apparent extinction coefficient.

#### (iv) Pigment particles on liquid surfaces and after agitation

The immersion test was carried out by placing 50 mg of each pigment powder carefully on the surface of either 3 cm^3^ of water or *n*-heptane in a screw cap glass vessel (internal diameter, 1.8 cm; height, 7.2 cm). We observed if the particles entered into the liquids or not. After 2 h, the mixtures were vigorously shaken for 30 s at room temperature.^28,30^ Photographs of the vessels were taken before and after agitation using a Canon camera IXUS 170.

#### (v) Preparation, stability and characterization of emulsions

The powdered particle method was employed for the preparation of emulsions.^31^ Equal volumes (5 cm^3^) of water and oil were used in 14 cm^3^ screw cap vessels (internal diameter, 1.8 cm; height, 7.2 cm) for particle concentration scans while different volumes of water and oil were used when varying the volume fraction of water *φ*_w_ at fixed particle concentration. The oil–water–particle mixture was homogenized with an IKA Digital Ultra Turrax T25 homogenizer with 8 mm head operating at 12 000 rpm for 2 min. Immediately after emulsion preparation, the emulsion type was inferred by the drop test. Oil continuous emulsions dispersed in oil and remained as drops in water and *vice versa*. The conductivity of the emulsions was determined using a Jenway 4310 digital conductivity meter equipped with Pt/Pt black electrodes. The fraction of oil or water released from emulsions with time is calculated by dividing the volume of oil or water separating by the total volume of oil or water used initially. The stability of oil-in-water (o/w) emulsions to creaming was assessed by monitoring the increase with time of the position of the clear water (serum)–emulsion interface, whereas the extent of coalescence was estimated from the movement of the oil–emulsion boundary. For water-in-oil (w/o) emulsions, the downward movement of the oil–emulsion boundary was used as a measure of the stability to sedimentation, and the position of the water–emulsion interface was used as an indicator of coalescence.^28^ Photographs of the vessels containing emulsions were taken with a Canon camera IXUS 170.

The morphology of the emulsions was investigated using an Olympus BX51 optical microscope fitted with CCD camera system DP70. The samples were taken from the middle of the emulsion layer. They were diluted around 50% with the continuous phase. The images were processed with Image Pro Plus 6.0 software. The average droplet sizes of the emulsions were measured with ImageJ. The size of the non-spherical drops was determined by Martin's diameter measurement method.^32^ This was done by finding the length of the line that bisects the image of the droplets in a given direction. The line may be drawn in any direction but the direction must be maintained constant for all the measurements.

For cryo-SEM analysis, 50 μL of emulsion was applied to an Al sample mount which was then plunged into liquid nitrogen at –170 °C. Using a transfer rod, the sample was placed into the preparation chamber stage held at –140 °C (PP3010T Quorum Technologies Ltd.). It was then fractured and the freshly exposed surface was coated with 2 nm of Pt using a sputter coater. It was transferred into a Zeiss EVO 60 SEM stage for viewing at –140 °C.

## Results and discussion

### Estimation of pigment surface energy

The direct estimation of the surface energy of particles is still an unresolved problem. The indirect methods available have their advantages and limitations. The various methods used are based on the nature of the interactions between liquids and the solid surface, such as dispersive interactions^33,34^ or acid–base interactions.^35,36^ For simplicity, the surface energy of a solid material may be divided into a polar component, *γ* ^p^, and a dispersion component, *γ* ^d^. As described earlier,^37,38^ the contact angle *θ*_la_ of a liquid (l) drop in air (a) on a perfectly smooth and homogeneous surface (s) using the Young equation can be written in terms of the polar and dispersion components of the surface energies of the liquid and solid as1
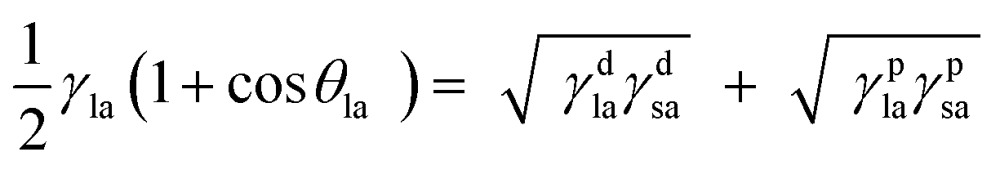



**Fig. 2:**
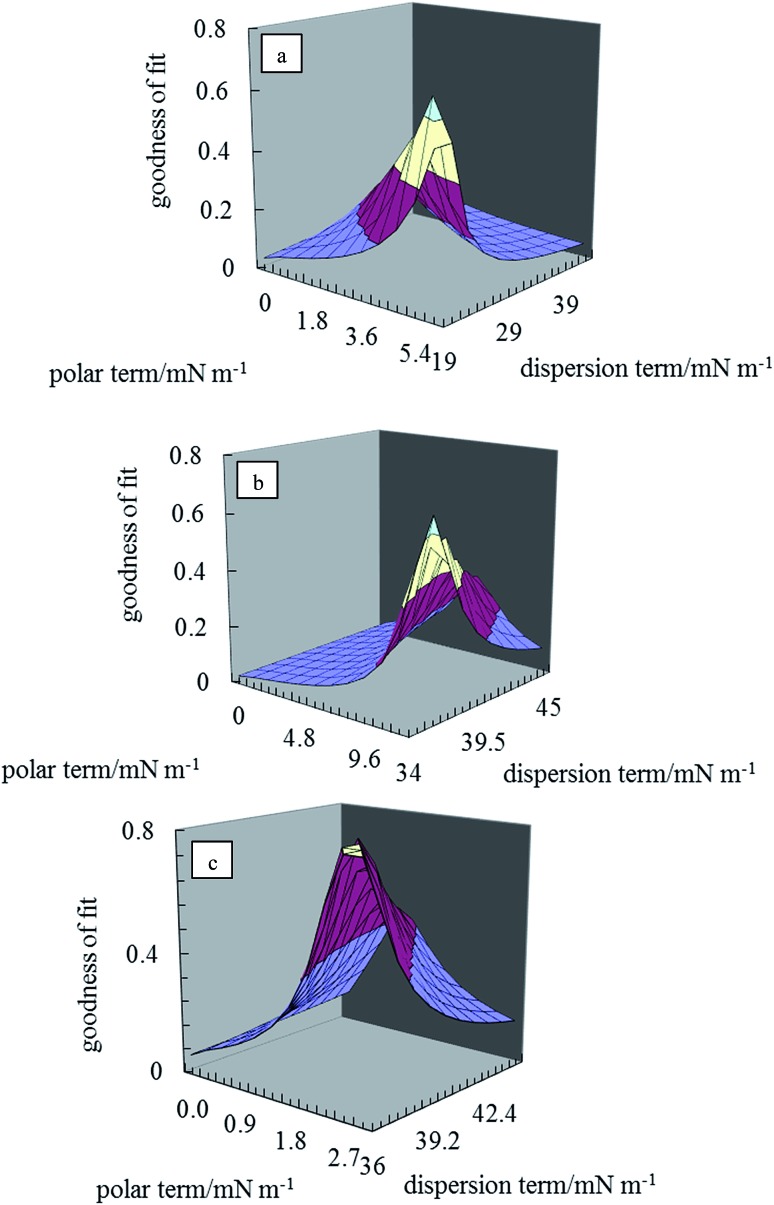
3-D surface energy plots for disks of the pigment particles for different values of *γ*dsa and *γ*psa for pigments (a) PR, (b) PO, (c) PV.

The two unknowns in eqn (1) are *γ*dsa and *γ*psa, and can be determined by solving the equation simultaneously. Values of the terms for the liquid in this equation (*γ*dla and *γ*pla) are given in Table 2 and *θ*_la_ is measured here on disks of the pigment particles. Literature reports show that it is advisable to use more than two liquids of different polarity to estimate the surface energy.^35,39^ For this purpose, water, glycerol, formamide, α-bromonaphthalene and *n*-hexadecane were used. The least squares calculation was carried out to determine the best combination of *γ*dsa and *γ*psa that fits all the data simultaneously. The 3-D surface energy diagram so obtained is shown in Fig. 2 for pigments PR, PO and PV. Those for the remaining pigments are displayed in Fig. S3.† This is a plot of the goodness of fit to contact angle set against a matrix of possible values of *γ*dsa and *γ*psa. The value that best fits all the contact angles is read from the coordinates that defines the peak in the chart.^39^ These values are given in Table 3. The estimation in this way of the surface energy of silica,^39^ fluorinated clay,^38^ inorganic pigment^40^ and some organic pigments^41^ has been reported. For so-called hydrophilic Crown glass,^42^ the surface energy was estimated to be 76 mN m^–1^ while the surface energy for a very hydrophobic surface like PTFE^43^ was estimated to be 18 mN m^–1^. Lim *et al.* ^40^ showed that the surface energy of titania pigments coated to different extents with silane reagent decreases (*i.e*. hydrophobicity increases) as the degree of surface coating increases. From Table 3, it can be seen that pigment PO is the most hydrophilic with a high polar contribution. On the other hand, pigment PY is the most hydrophobic with a very small polar contribution. All the pigments have a higher value for the dispersion component than the polar component. The seven organic pigments used here can be grouped into three; pigments PR, PY, PG and PB have relatively low surface energy, pigments PI and PV have intermediate surface energy whilst pigment PO has relatively high surface energy.

**Table 2:** Values of dispersion component, polar component and *γ*_la_ for the liquids used at 25 °C (taken from ref. 43) and both the three-phase equilibrium advancing liquid–air contact angles and the oil–water contact angles measured on the surface of disks formed from the pigment particles

Liquid	Tension/mN m^–1^			Liquid–air contact angle/°						
	*γ* d la	*γ* p la	*γ*_la_	PR	PO	PY	PG	PB	PI	PV
Water	21.6	50.5	72.1	87	64	108	92	93	80	85
Glycerol	34.0	30.0	64.0	74	44	100	77	79	72	66
Formamide	39.0	19.0	58.0	72	37	83	75	78	53	58
α-Bromonaphthalene	44.4	0.0	44.4	<5	<5	<5	<5	<5	<5	<5
*n*-Hexadecane	27.8	0.0	27.8	<5	<5	<5	<5	<5	<5	<5

	Oil–water contact angle/°						
	PR	PO	PY	PG	PB	PI	PV
Heptane–water (expt.)	143	84	142	144	139	141	143
Heptane–water (calc.)	110	92	146	125	124	112	122

**Table 3:** Surface energy for the seven pigments as well as their corresponding polar and dispersion components

Pigment	*γ* d sa /mN m^–1^	*γ* p sa /mN m^–1^	*γ*_sa_/mN m^–1^
PR	21.5	5.4	26.9 ± 0.3
PO	36.2	12.0	48.2 ± 0.7
PY	20.2	0.2	20.4 ± 0.3
PG	27.2	2.0	29.2 ± 0.3
PB	25.1	2.0	27.1 ± 0.2
PI	34.0	3.6	37.6 ± 0.4
PV	36.0	2.7	38.7 ± 0.4

### Solubility of pigments in water or *n*-heptane

**Fig. 3:**
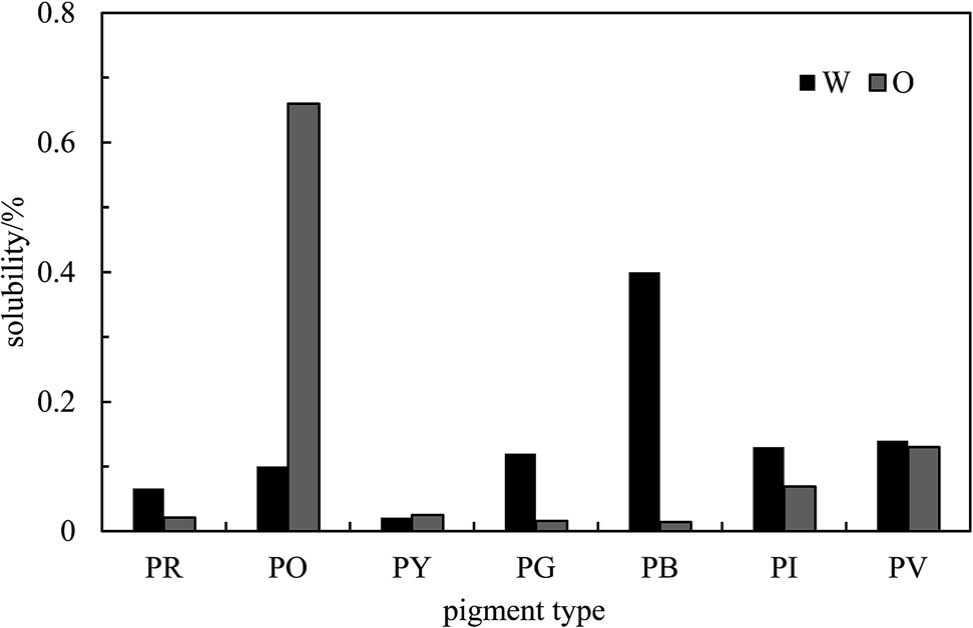
Solubility of the seven pigments in water (W) and *n*-heptane (O) determined by spectrophotometry at room temperature.

The absorption spectrum obtained for each pigment in water or *n*-heptane^44^ is given in Fig. S4.† The values of *λ*_max_ obtained for the unfiltered dispersion and the supernatant are summarized in Table S2.† The graphs of absorbance against concentration for unfiltered dispersions are displayed in Fig. S5† for each pigment. Non-zero intercepts are due to the slight absorbance of neat solvent in some cases or due to slight imperfections in the cuvettes. Values of the apparent extinction coefficients deduced from the slopes of these plots are given in Table S3.† Knowing these and the absorbance of the supernatant after filtering, the solubility of the pigments in the two solvents can be calculated and they are shown in Fig. 3. As expected, these solubilities are very low and ≤0.1% in most cases in both solvents. We verified that no stable emulsion could be prepared from solutions of the pigments at their solubility limits such that emulsion stabilization at concentrations above this is due entirely to the presence of pigment particles.

### Behaviour of pigment particles on liquid surfaces

**Fig. 4:**
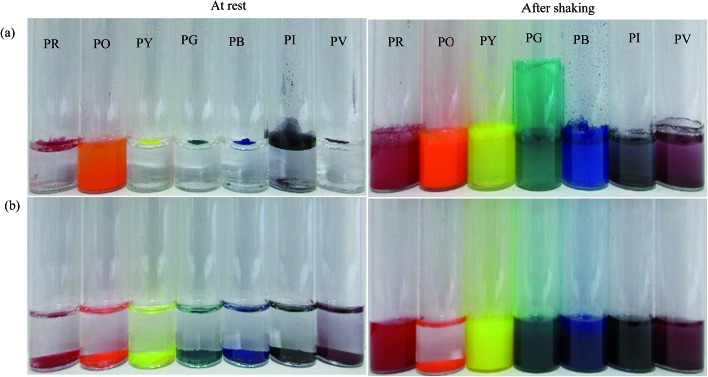
Photos of vessels showing the appearance of a mixture of 50 mg of pigment particles and 3 cm^3^ of either (a) water or (b) *n*-heptane before and after agitation for 30 s.

In order to assess how the pigments interact with either water or oil, 50 mg of each pigment was added to the surface of 3 cm^3^ of the liquids as shown in Fig. 4. At rest, only the most hydrophilic pigment PO is wetted by water spontaneously forming a turbid dispersion whilst the others remain on its surface. By contrast, all of the pigments are spontaneously wetted by *n*-heptane and sediment. It appears that pigment PO is omniphilic because it has a high affinity for both liquids due to its relatively high surface energy.^38^ After agitation in both solvents, turbid dispersions with sediment were observed for all the pigments except PO in *n*-heptane which formed a clear supernatant with some particles suspended at the liquid surface. In water, climbing films^30^ were also observed for pigments PG, PI and PV while unstable foams were formed by pigments PR, PY and PB. Climbing films are formed when air bubbles sparsely coated with particles coalesce with the planar air–water surface releasing their particles, which causes an increase in the concentration of adsorbed particles leading to an increase in surface pressure causing the film to move upward. Available literature results show that climbing films and foams are an indication of the behavior of reasonably hydrophobic particles when agitated in water, while a mixture of a low surface tension liquid and relatively high surface energy particles produce only a dispersion.^30,39^

### *n*-Heptane–water emulsions stabilized by pigment particles

#### (i) Effect of particle concentration for *φ*_w_ = 0.5

**Fig. 5:**
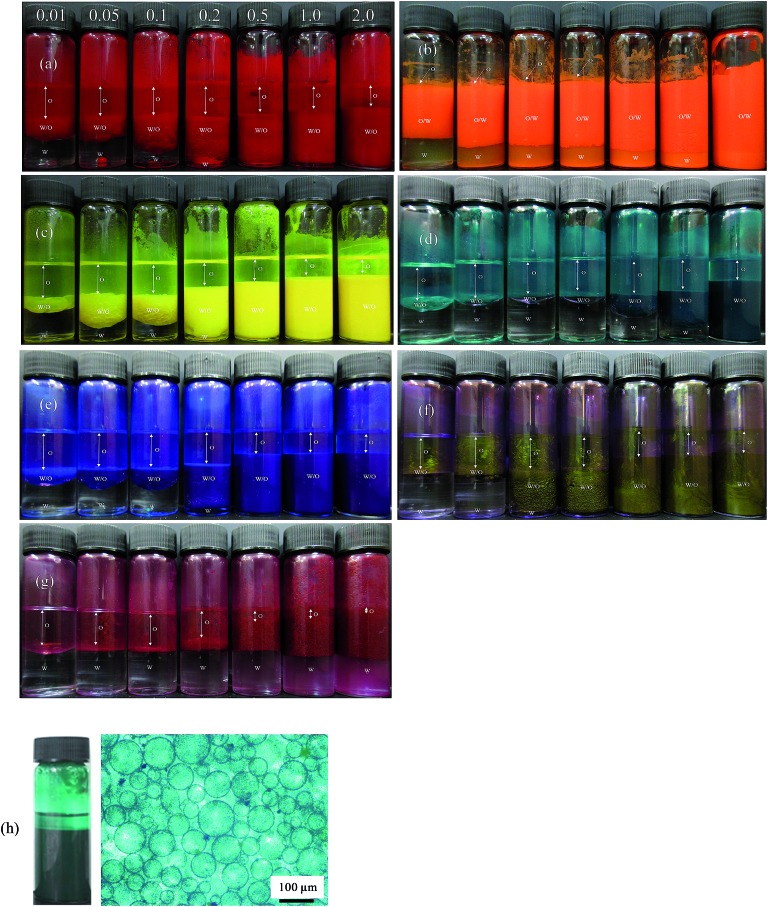
Photographs of emulsions after 1 month prepared at different particle concentrations (given in wt%) for the seven pigments with *φ*_w_ = 0.5: (a) PR, (b) PO, (c) PY, (d) PG, (e) PB, (f) PI, (g) PV. Emulsions are w/o except those of PO which are o/w. In (h), w/o emulsion and microscope image of the mixture containing 0.5 wt% each of PY and PB.

Emulsions were prepared using the powdered particle method at a volume fraction of water *φ*_w_ = 0.5 as a function of particle concentration. Photographs of the vessels containing the emulsions one month after preparation are given in Fig. 5. For pigment PO, emulsions were o/w at all concentrations whereas they were water-in-oil (w/o) for all the other pigments. This is consistent with the order of particle surface energies discussed above. In particle-stabilised emulsions, the emulsion type is related to the relative wettability of particles by oil and water which can be gauged by the oil–water contact angle.^28^ Applying the appropriate form of eqn (1), the oil–water contact angle can be calculated for the different pigments using the surface energies of the pigments estimated earlier. These values along with the measured contact angles on particle disks are given in Table 2 where it can be seen that agreement is reasonable for most pigments. Importantly, the experimental oil–water contact angle for pigment PO is <90° implying the preferred emulsion is o/w while that for the other pigments is >90° implying preference for w/o emulsions. The results obtained here are in good agreement with previous work for other particle types.^42^

**Fig. 6:**
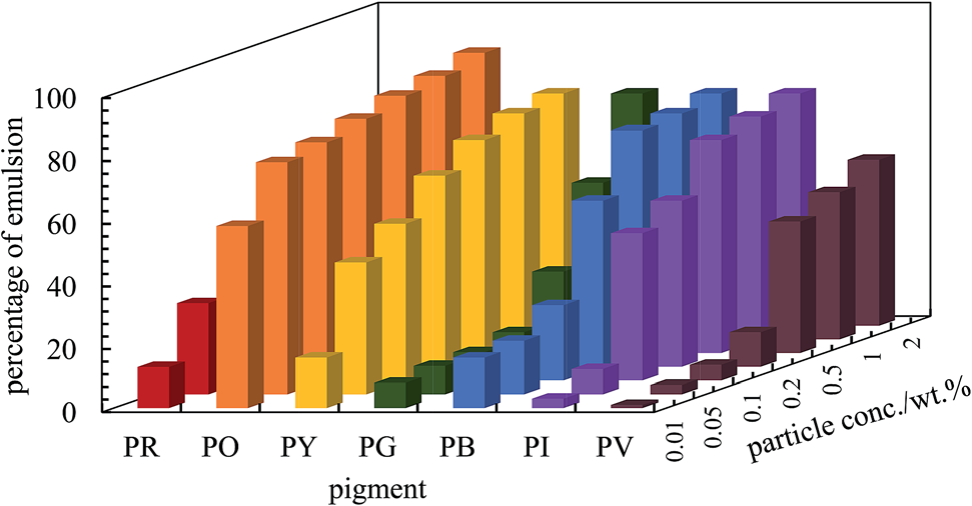
Percentage of emulsion remaining after three months for the seven pigments at various particle concentrations for *φ*_w_ = 0.5. Emulsions are w/o except those of PO which are o/w.

For PO, most of the o/w emulsions cream resolving a water phase below (w), and above 0.1 wt% particles no oil (o) is seen above the cream since emulsions become stable to coalescence. For the w/o emulsions, sedimentation occurs to different extents liberating an upper oil phase and those stabilized by pigments PR, PY, PB and PI become very stable to coalescence at higher particle concentrations. Those w/o emulsions less stable to coalescence are with pigments PG and PV. The percentage of emulsion (e) remaining after 3 months (vol emulsion/total vol oil and water) is represented by the bar chart in Fig. 6. This percentage increases as the particle concentration increases for all the pigments, although the change varies from one pigment to the other. Unlike the w/o emulsions, the o/w emulsions of PO are quite viscous. The stability of Pickering emulsions depends on a variety of factors including particle concentration,^45^ pH^46^ and salt concentration.^47^ Fig. S6† shows the time course for the release of both oil (due to sedimentation if w/o and coalescence if o/w) and water (due to coalescence if w/o and creaming if o/w) from emulsions prepared at 1 wt% particles. In the majority of systems, the changes occur within the first 5 min although some emulsions (notably of PG and PV) continue to evolve subsequent to this.

**Fig. 7:**
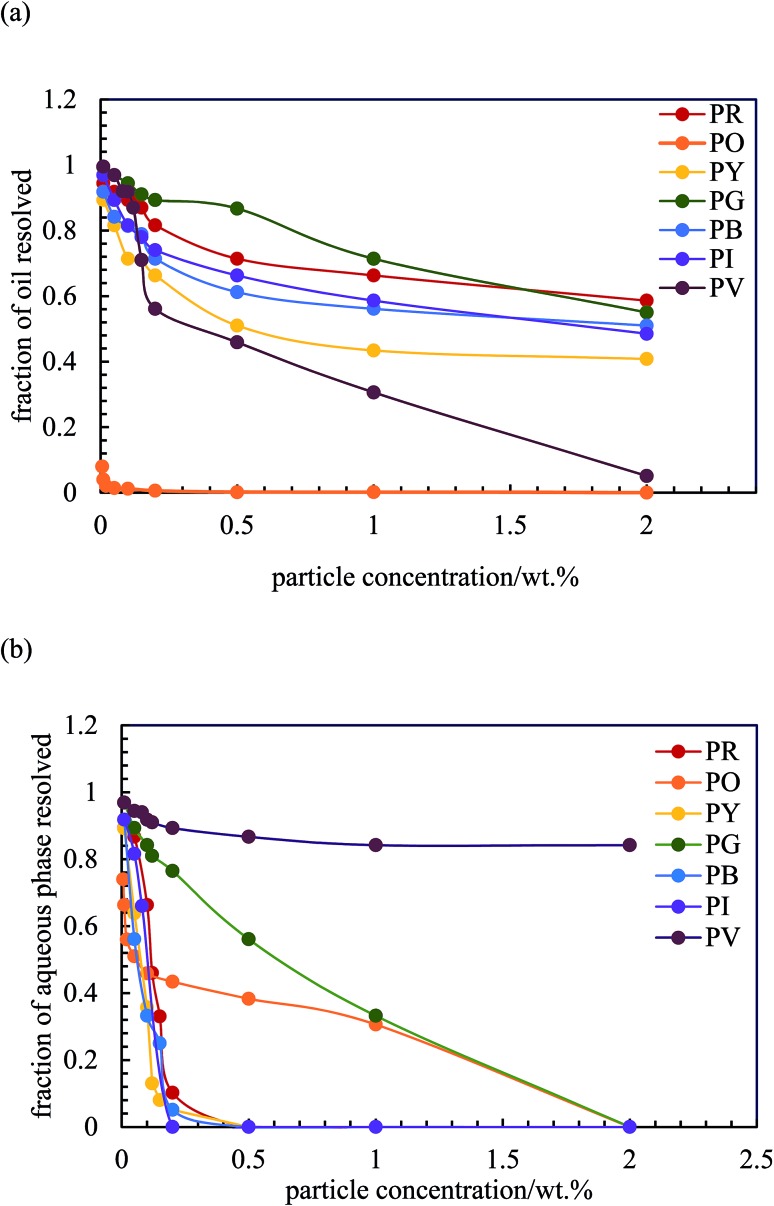
Fraction of (a) oil and (b) aqueous phase resolved after three months from emulsions stabilized by the seven pigments, *φ*_w_ = 0.5, as a function of particle concentration.

After 3 months, no further change in emulsion stability was detected and the fraction of oil or water resolved at this time is plotted as a function of particle concentration in Fig. 7. For all emulsions, these values decrease with particle concentration. As the pigment concentration is increased, more particles are available to cover the droplet interfaces, thereby preventing them from coalescing. At low pigment concentration, the droplets are partially covered with particles and they coalesce accounting for the large fraction of aqueous phase resolved for w/o emulsions. Apart from PG and PV-stabilised emulsions, only 0.5 wt% of particles is required to yield emulsions stable to coalescence. For pigment PG, this requires 2 wt% particles whereas for pigment PV coalescence is appreciable even at this higher concentration. The different degrees of stability observed in emulsions stabilized by the seven pigments may be due to differences in the size,^48^ shape^49^ and surface energy of the particles.^46^

**Fig. 8:**
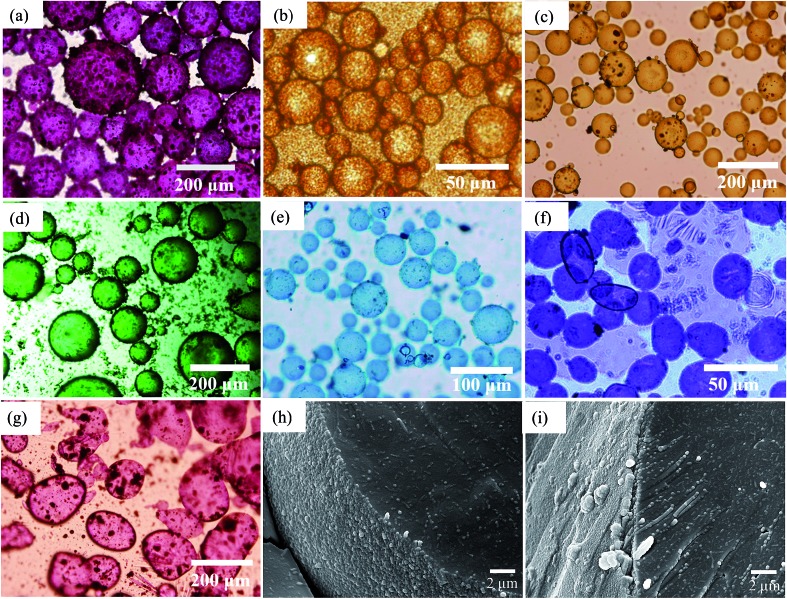
Optical microscope images of emulsions taken after 20 min for *φ*_w_ = 0.5 prepared at 1 wt% particles and the seven pigments for (a) PR, (b) PO, (c) PY, (d) PG, (e) PB, (f) PI, (g) PV. Emulsions are w/o except those of PO which are o/w. Cryo-SEM images for emulsions of (h) PY and (i) PR.

As an example, optical micrographs of emulsions prepared with 1 wt% of particles are given in Fig. 8(a)–(g), while those for other concentrations are displayed in Fig. S7.† Larger particles can be seen on the surface of some of these droplets, particularly for pigments PR, PG, PB and PV. The influence of particle concentration on average drop size has been studied using different colloidal particles like silica,^31^ protein zein^46^ and microgels.^50^ Generally, it is found that the drop size depends on both the particle concentration and on the speed of homogenization. Here, we found that the average droplet size also decreased with particle concentration up to a limit. This occurs because there are more pigment particles available to stabilize smaller droplets and their accumulation at the oil–water interfaces provides mechanical support against coalescence.^51,52^ For o/w emulsions of PO which form the smallest droplets, these are spherical and polydisperse and appear slightly flocculated at all concentrations studied. For w/o emulsions, droplets are polydisperse, discrete and of spherical morphology except those of PI and PV whose droplets are distinctly non-spherical. For the latter, droplets with different geometries such as ellipsoidal, sphero-cylindrical and irregular shape were formed at low pigment concentrations (0.01–0.2 wt%), whilst at higher concentrations (>0.2 wt%) both spherical and ellipsoidal droplets were predominantly formed (Fig. S7†). Non-spherical droplets are a result of either jamming of the interfacially trapped particles,^53,54^ or the buckling and crumpling of the particle layer.^55^ Both scenarios prevent relaxation of the droplet to a spherical geometry.^56^

We have analysed the dependence of droplet size on particle concentration in the framework of the phenomenon of limited coalescence.^57^ This process takes place in a system emulsified at low particle concentration. After homogenization is complete, the formed droplets coalesce because their interfaces are only partially covered by particles and this is accompanied with a reduction in the total interfacial area. Since particles are adsorbed irreversibly at the interface, droplet coalescence is halted as soon as the interfaces are sufficiently covered by particles. The amount of particles available determines the final droplet surface area and their packing at interfaces. Assuming all particles become adsorbed, the surface coverage C may be defined as the ratio of the interfacial area, *S*_a_, which could be covered by spherical particles taking into account their size and the total interfacial area, *S*_c_, equal to 6*V*_d_/*D* where *V*_d_ is the volume of disperse phase and *D* is the drop diameter. This provides information about the percentage of the droplet surface area covered by the particles which links to the arrangement of the particles at the droplet interface. According to previous studies, particles adsorb either forming a dense monolayer^57^ or a multilayer^58^ but cases where the interfacial coverage was surprisingly low have also been reported.^59,60^ For a hexagonal close-packed monolayer of monodisperse particles, *C* should be equal to around 0.9. If the surface coverage is <0.9, this implies that the particles are not close packed at the interface while a larger value suggests the formation of a multilayer or aggregates at the interface.^60^ It can be shown that2
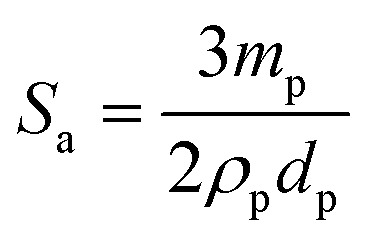
where *ρ*_p_ is the particle density (g cm^–3^) and *d*_p_ is the particle diameter (μm). Thus,3
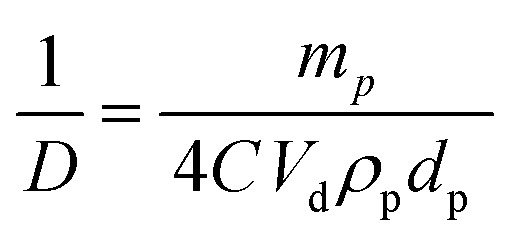



 Eqn (3) suggests that a plot of the inverse of droplet diameter against the mass of the particles, *m*_p_, should be linear allowing C to be determined from the slope as the other parameters for each pigment are known.

**Fig. 9:**
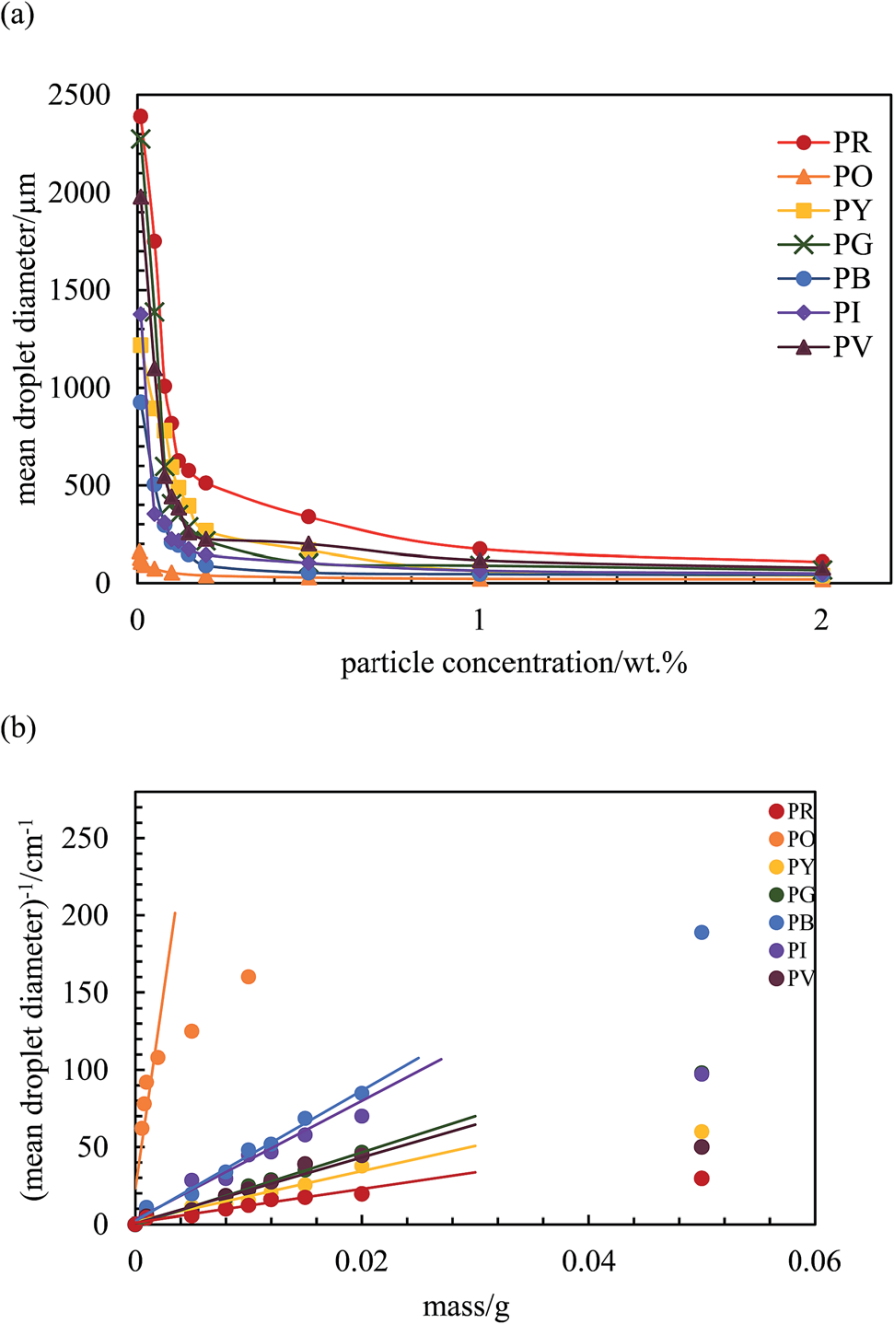
(a) Variation of the mean droplet diameter after 3 months with particle concentration for the seven pigment-stabilized emulsions, *φ*_w_ = 0.5, (b) plot of inverse of mean droplet diameter against initial mass of particles for emulsions in (a).

The variation of final droplet size with particle concentration for all the pigment-stabilised emulsions is shown in Fig. 9(a). Droplets at low concentration larger than 1000 μm are visible to the eye and we note the particularly small sizes in PO-stabilised emulsions. As expected, the droplet size reaches a limiting value above around 0.5–1.0 wt% particles. The inverse of the droplet diameter is plotted against the particle mass in Fig. 9(b). The linear variation at low concentrations testifies that limited coalescence takes place as the origin of the final droplet size. The surface coverage *C* for each pigment-stabilized emulsion is given in Table 4. We would like to point out that the way particles are attached to the interface remains unknown in many cases, and that adsorption of aggregates or clusters may occur. If so, clusters are attached to interfaces through a reduced number of anchoring particles and that other particles in the clusters protrude towards the continuous phase. The surface coverage obtained for pigments PR, PY and PV is greater than 0.9. Consistent with this, cryo-SEM images of water drops given in Fig. 8(h) and (i) reveal the presence of a single monolayer in the case of PY (lower *C* value) but >1 layer in the case of PR (highest *C* value). Importantly, primary pigment particles of size comparable to that in the powder adsorb at the interface implying that high shear homogenisation is sufficient to disrupt aggregates, in the absence of a dispersant. Emulsions of pigments PG and PI have a surface coverage of <0.9 suggesting that the particles are more loosely packed at the interface. Finally, emulsions of PB and particularly PO have low values of *C*. By drying the separated oil (for w/o) and water (for o/w) from emulsions to constant weight, we determined that at 0.2 wt% particles more than 99 ± 1% of particles were associated with droplets for all w/o emulsions whereas only 90 ± 1% of particles were adsorbed around oil drops in the PO-stabilised emulsion. This can also be appreciated by the micrographs for PO in Fig. S7† revealing many particles in the continuous aqueous phase; we recall from earlier that this pigment disperses spontaneously in both solvents. Application of eqn (3) in the latter case is then not straightforward since the percentage of non-adsorbed particles may depend on initial particle concentration. We suggest that particles on oil droplet interfaces are contiguous with those in bulk water such that a network of particles contributes to emulsion stability. Long term stabilization of emulsions in cases where the coverage *C* is low has been reported in several cases for both o/w and w/o emulsions.^61–63^ More recently,^64^ o/w emulsions exhibiting low surface coverage of rod-shaped cellulose particles has been reported. Depending on the rod length, *C* values between 44% and 84% were found to be required for emulsion stabilization. It appears to occur under conditions where particles are charged and exhibit mutual repulsion. Interfacial particle clusters separated by areas of bare interface is common. Consistent with this, the zeta potential of PO particles hand shaken into water (5 mg L^–1^) was – 22 ± 2 mV.

**Table 4:** Surface coverage *C* determined for emulsions stabilized by pigment particles, *φ*_w_ = 0.5

Pigment	*C* (±0.10)
PR	1.71
PO	0.05
PY	1.00
PG	0.62
PB	0.17
PI	0.67
PV	1.20

It is interesting to ask what colour an emulsion will be when stabilised by a mixture of two pigments. As a proof-of-concept, we prepared an emulsion with *φ*_w_ = 0.5 containing 0.5 wt% each of PY and PB which yield stable emulsions when present alone. Reassuringly, as seen in Fig. 5(h), the emulsion is w/o (as for each pigment alone) and appears green being stable to coalescence even though it sediments. We note that the mixed powder before addition of either liquid is also green, implying that water droplets are coated by both particle types. We are continuing this and related studies with pigment mixtures to be reported in due course.

#### (ii) Effect of oil : water ratio for 1 wt% particles

**Fig. 10:**
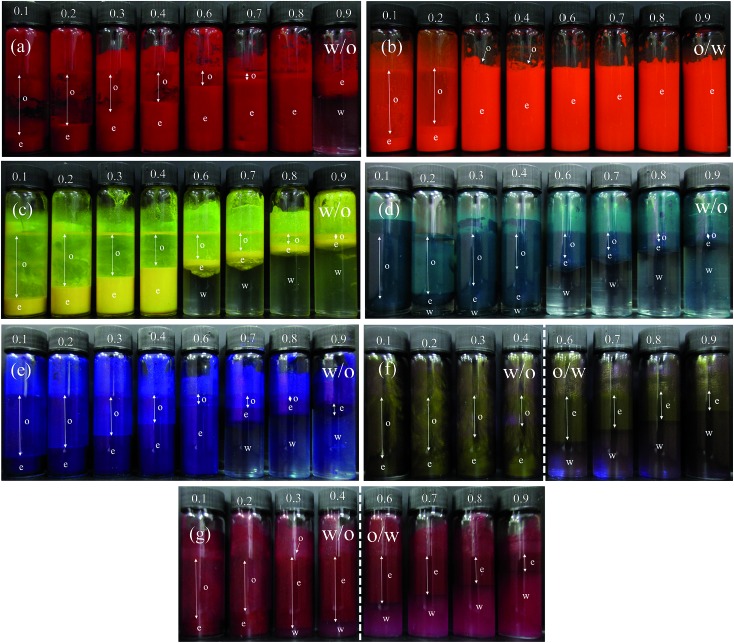
Photos of vessels after 30 min containing emulsions stabilized by 1 wt% of pigment particles at different values of *φ*_w_ (given) for (a) PR, (b) PO, (c) PY, (d) PG, (e) PB, (f) PI, (g) PV. The dotted line in (f) and (g) signifies catastrophic phase inversion.

**Fig. 11:**
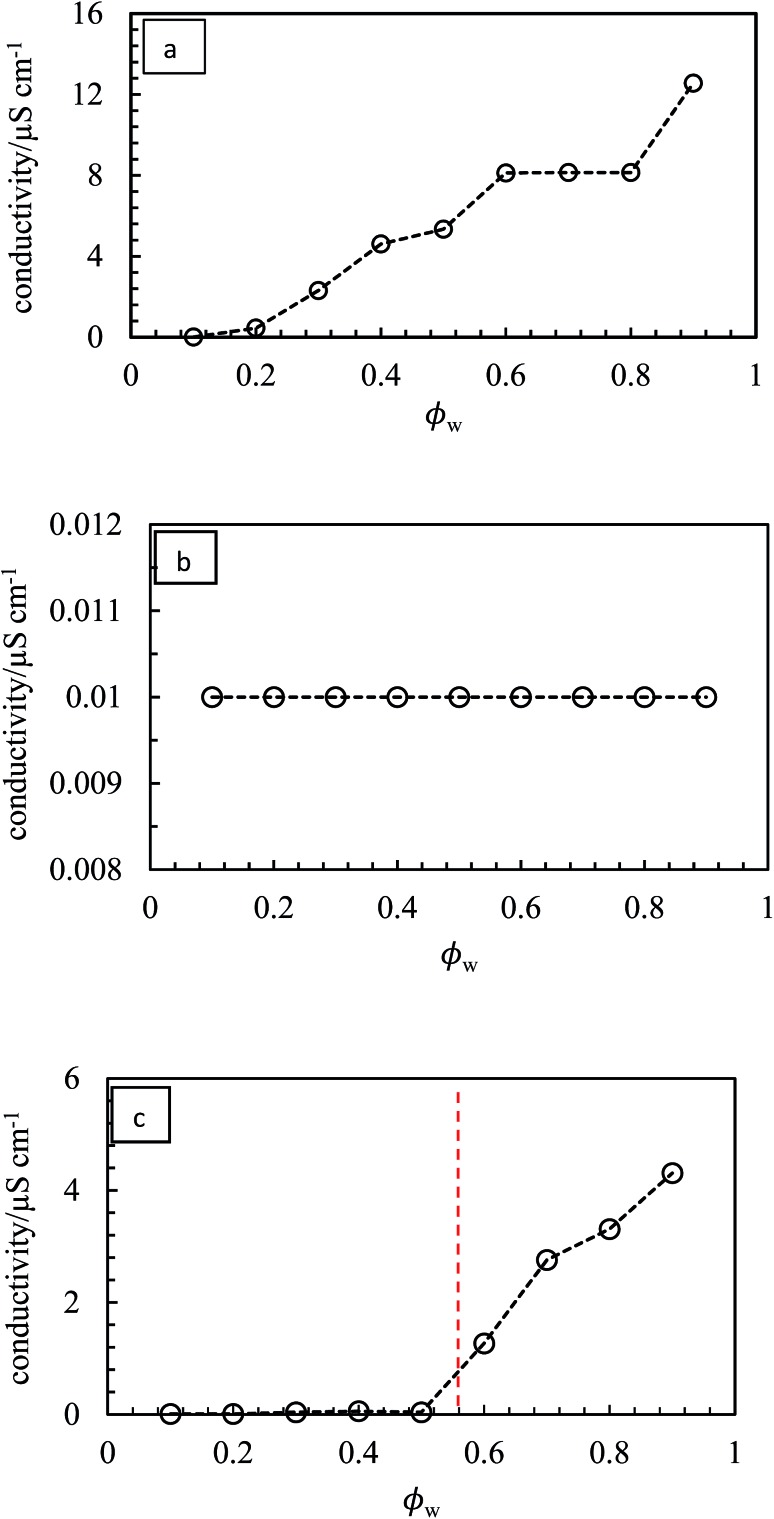
Variation of emulsion conductivity as a function of *φ*_w_ for the emulsions in Fig. 10 for (a) PO – o/w, (b) PG – w/o, (c) PV – w/o to o/w. The vertical dotted line signifies catastrophic phase inversion.

As seen above, preferred emulsions at equal volumes of oil and water are w/o for six of the pigments and o/w for one of them. In one and the same system, the possibility exists however that emulsion phase inversion can be effected by increasing the volume fraction of disperse phase, so-called catastrophic inversion.^65^ We chose a particle concentration of 1 wt% for which emulsions were stable to coalescence at *φ*_w_ = 0.5 and investigated the type and stability of emulsions formed at different oil : water ratios. Photographs of the vessels containing emulsions prepared at different aqueous phase volume fractions *φ*_w_ from 0.1 to 0.9 are shown in Fig. 10. We find three types of behavior, confirmed by both the drop test and measurement of emulsion conductivity (conductivity of water and heptane at 20 °C were 0.70 and 0.01 μS cm^–1^ respectively) shown in Fig. 11 and S8.† For pigment PO, emulsions were o/w at all values of *φ*_w_ with their conductivity increasing with water content (Fig. 10(b) and 11(a)). For pigments PR, PY, PG and PB, emulsions were w/o at all values of *φ*_w_ with conductivities similar to pure heptane (Fig. 10(a), (c)–(e), 11(b) and S8(a)–(c)†). For pigments PI and PV however, catastrophic phase inversion occurs upon increasing *φ*_w_ with emulsions inverting from w/o to o/w and the conductivity exhibiting a significant increase around inversion at *φ*_w_ ≈ 0.55 (Fig. 10(f), (g), 11(c) and S8(d)†). The pattern of behavior is reminiscent of that found earlier for emulsions stabilized by fumed silica particles of different wettability.^45^ Emulsions of the most hydrophilic pigment PO cannot be inverted, as can't those of the most hydrophobic pigments PR, PY, PG and PB. Emulsions stabilized by particles of intermediate hydrophobicity like PI and PV however are sensitive to prevailing conditions and can be phase inverted. This is an important finding, *i.e*. both emulsion types can be stabilized by one and the same particle type.

**Fig. 12:**
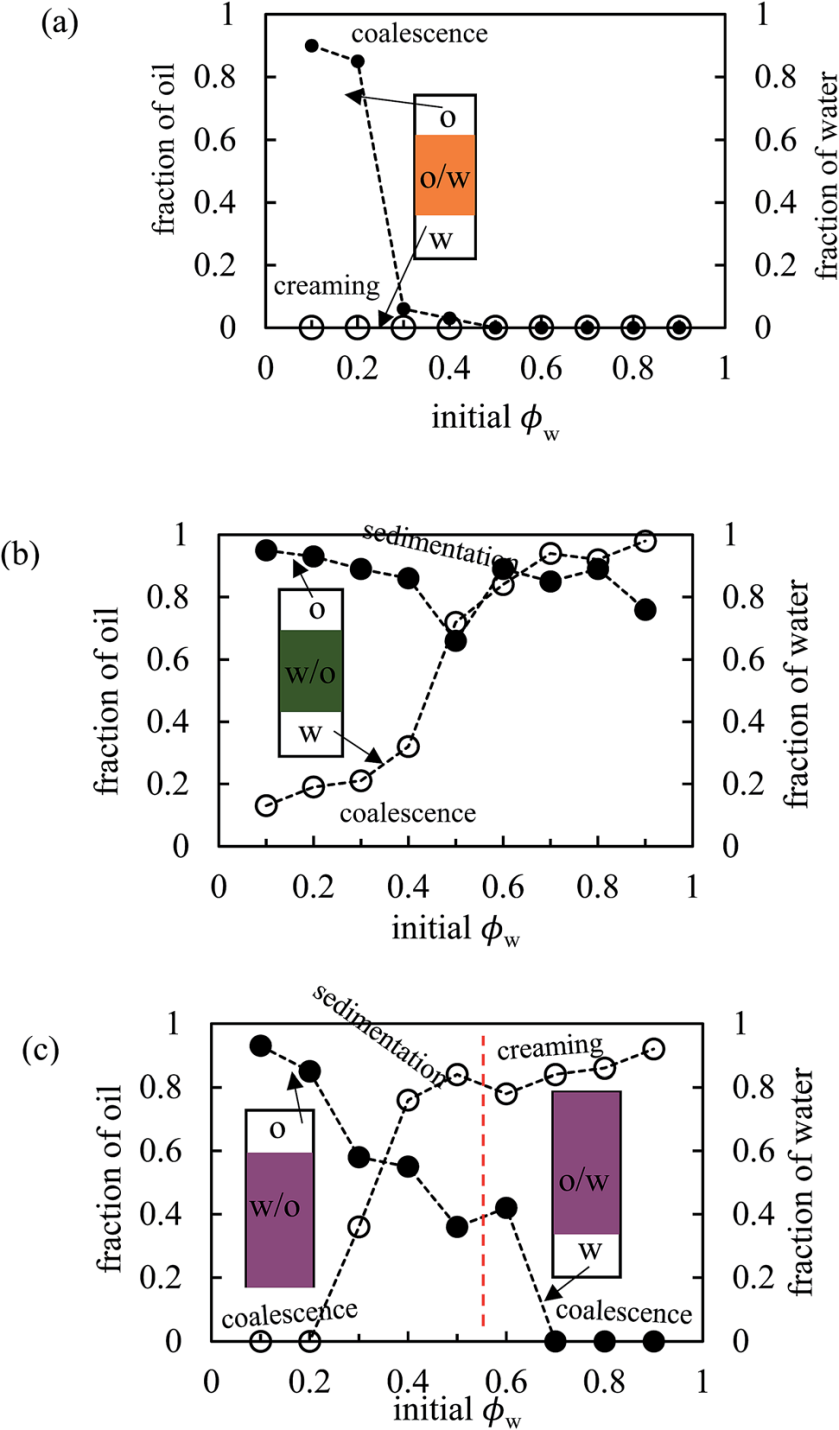
Variation of fraction of oil (filled points) and water (open points) resolved after two months as a function of initial water volume fraction for emulsions in Fig. 10 for (a) PO, (b) PG, (c) PV.

The stability of all the emulsions was monitored with time. We noticed that the various breakdown processes of creaming, sedimentation and coalescence occur within 30 min or so after emulsification. The stability at long time is plotted in terms of the fraction of oil and water released in Fig. 12 for emulsions of PO, PG and PV being representative of the three kinds of system (o/w, w/o and w/o→o/w respectively). Those for the other pigments are given in Fig. S9.† Oil-in-water emulsions of PO, Fig. 12(a), are stable to both creaming and coalescence at *φ*_w_ values of 0.5 and higher, *i.e*. for disperse oil phase volume fractions up to 0.5. As the droplet volume fraction increases further, coalescence sets in progressively such that at *φ*_w_ = 0.2 only a small volume of emulsion can be stabilized. In w/o emulsions of PG, Fig. 12(b), the extent of coalescence increases progressively with an increase in *φ*_w_, whilst the extent of sedimentation remains high throughout. For the other non-inverting w/o emulsions of PR, PY and PB (Fig. S9†), excellent stability to coalescence is achieved up to drop volume fractions of 0.8, 0.5 and 0.6 respectively, above which coalescence occurs. It is worth pointing out that since these emulsions also exhibit sedimentation, the volume fraction of water drops in stable emulsions will be higher than the initial value of *φ*_w_ such that high internal phase emulsions^66,67^ can form which remain stable due to particle-coated droplet interfaces. Finally, for emulsions of PV which phase invert, Fig. 12(c) shows that w/o emulsions at low *φ*_w_ coalesce progressively towards phase inversion, whilst the extent of sedimentation decreases. After inversion, o/w emulsions become completely stable to coalescence at oil droplet volume fractions of ≤0.3 and the stability to creaming decreases. A similar trend is seen in inverting emulsions of PI (Fig. S9(d)†), although they are more stable to coalescence overall.

**Fig. 13:**
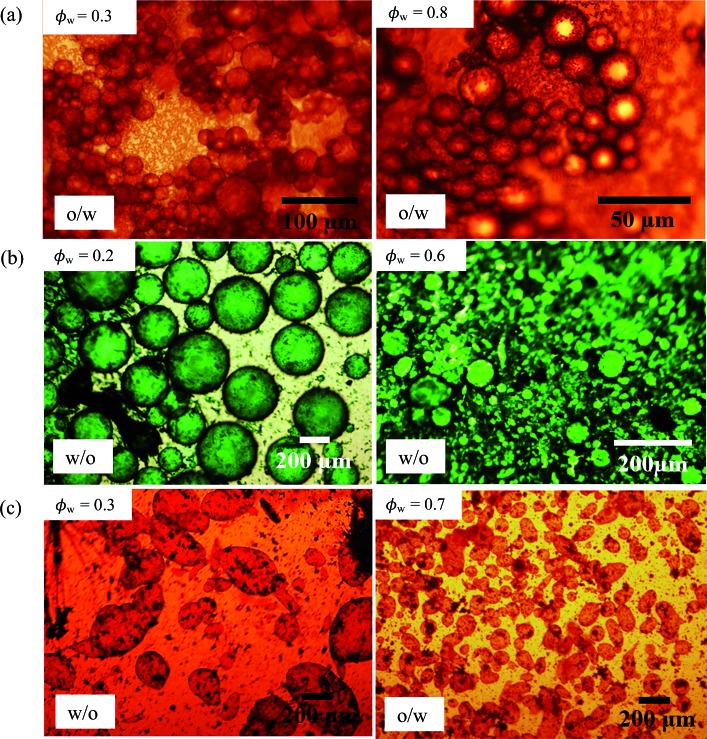
Optical micrographs of emulsions of Fig. 10 after 20 min at different *φ*_w_ (given) for (a) PO, (b) PG, (c) PV.

**Fig. 14:**
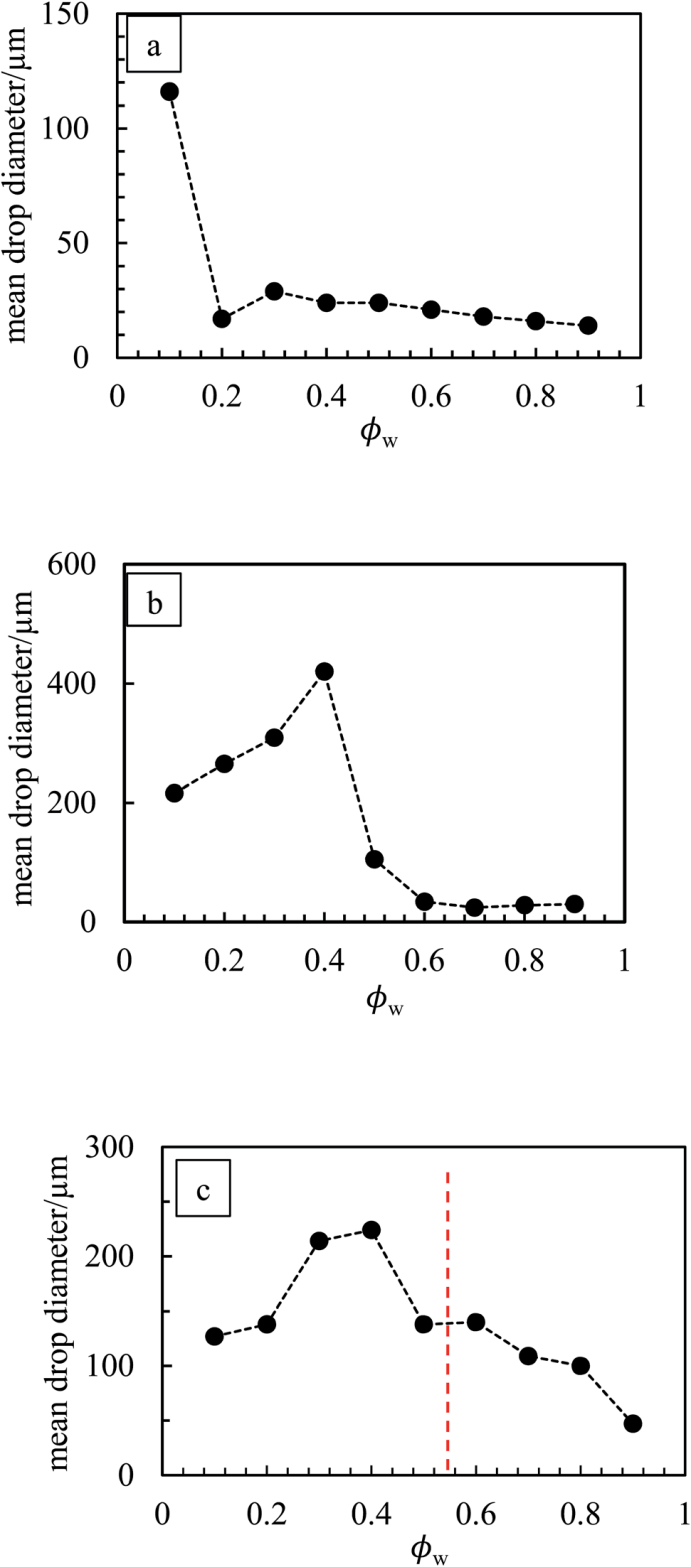
Variation of average drop diameter with *φ*_w_ for emulsions in Fig. 10 for (a) PO, (b) PG, (c) PV.

The marked decrease in the stability of emulsions to coalescence occurring at high droplet volume fraction may be because, at 1 wt% particles, insufficient particles are available to cover droplet interfaces. New emulsions were prepared at 3 wt% particles for PO (*φ*_w_ = 0.2), PR (*φ*_w_ = 0.9), PY (*φ*_w_ = 0.6 and 0.9), PG (*φ*_w_ = 0.7) and PB (*φ*_w_ = 0.8). In line with our prediction, all emulsions exhibited higher coalescence stability compared with those at 1 wt% particles. Optical micrographs of emulsions stabilized by 1 wt% of PO, PG and PV at different values of *φ*_w_ are shown in Fig. 13 while those of the other pigments are given in Fig. S10.† For PO, oil droplets remain spherical even at a high volume fraction (*φ*_w_ = 0.3), whereas for PG a proportion of w/o drops become non-spherical at high *φ*_w_, also evidenced for emulsions of PR, PY and PB. For emulsions of PI and PV which phase invert, the structure changes from being discrete water drops to more flocculated oil drops after inversion. Fig. 14 (and corresponding Fig. S11†) depicts the variation of the mean droplet diameter with the volume fraction of water. It increases steadily with drop volume fraction for o/w emulsions of PO until a large increase at *φ*_w_ = 0.1 due to coalescence, (a). For the non-inverting w/o emulsions (b), the diameter increases initially with increasing drop volume fraction and then decreases markedly at *φ*_w_ ≥ 0.5 depending on the pigment type. This occurs as drops assume non-spherical shapes. For emulsions which invert, (c), the drop diameter increases towards the inversion condition for both w/o and o/w emulsions consistent with an increase in the extent of coalescence. We note also that oil drops in general are smaller than water drops.^31,51^

#### (iii) Correlation between pigment surface energy and emulsion behaviour

**Fig. 15:**
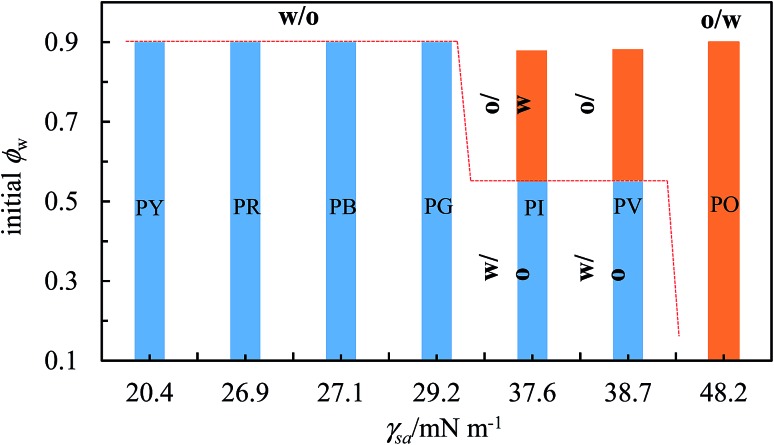
Correlation between transitional phase inversion (induced by a change in particle surface energy) and catastrophic phase inversion (induced by a change in *φ*_w_) of heptane–water emulsions stabilized by pigment particles of different type.

We have seen above that preferred emulsions at equal oil and water volume ratios are either o/w or w/o depending on the type of pigment, which is mostly due to its inherent surface energy. The most hydrophilic PO prefers to stabilize o/w emulsions whilst the more hydrophobic particles of the other pigments stabilize w/o emulsions. This difference in emulsion type with particle surface energy is an example of transitional phase inversion, evidenced previously for a range of fumed silica particles of increasing extent of silane coating.^45^ However, it does not allow us to distinguish the more hydrophobic pigments since they all yield w/o emulsions. The other type of phase inversion is catastrophic inversion brought about by varying the oil : water ratio in emulsions.^65^ Although the precise origin of this inversion is still unclear, one can argue that it should not be due to changes in the wettability of particles at the interface since all the components remain the same. It is linked in some way to the increase in droplet volume fraction approaching inversion. Now we are able to qualitatively grade the more hydrophobic pigments since those of intermediate surface energy stabilized emulsions which exhibited catastrophic phase inversion as opposed to those of highest surface energy which did not. We combine the two ideas in Fig. 15 by plotting the kind of emulsion which forms as a function of both the pigment surface energy (abscissa) and the value of *φ*_w_ (ordinate). We see that up to a surface energy *γ*_sa_ of around 30 mN m^–1^, the more hydrophobic pigments of PY, PR, PB and PG only stabilize w/o emulsions. For intermediate values of *γ*_sa_ between around 35 and 40 mN m^–1^, both emulsion types can be stabilized (pigments PI and PV). At higher values of *γ*_sa_ near 50 mN m^–1^, only o/w emulsions can be stabilized (pigment PO). We thus propose that the magnitude of *γ*_sa_ could be used as a guide to predict whether particle-stabilised emulsions are likely to exhibit catastrophic phase inversion or not. Although for the pigments we selected here there is not a linear variation in their surface energy, this could be effected by investigating a wider range of pigment types.

## Conclusions

Coloured organic pigment particles of different chemical structure can act as excellent Pickering stabilizers of heptane–water emulsions. Their solubility in both solvents is shown to be very low. Their relative surface energies may be determined from appropriate contact angle measurements on disks of the powders. The preferred emulsion type for equal oil and water volumes is o/w for the most hydrophilic pigment (PO) and w/o for the other more hydrophobic pigments (PR, PY, PG, PB, PI, PV). Such emulsions undergo limited coalescence to yield emulsions which do not coalesce with time at particle concentrations above around 1 wt%. The coverage of droplet interfaces by particles is dependent on the pigment type however. At constant particle concentration of 1 wt%, catastrophic phase inversion of emulsions from w/o to o/w occurs upon increasing *φ*_w_ for pigments PI and PV which exhibit intermediate values of surface energy. By contrast emulsions are either o/w for relatively hydrophilic PO or w/o for the most hydrophobic pigments PY, PR, PG and PB independent of *φ*_w_. We observe that the primary particles become adsorbed to droplet interfaces during homogenization such that no dispersant is required to break up aggregates. This is particularly important in an industrial context meaning that formulations containing pigments and interfaces may be simplified. Is the perceived colour different for formulations in which the particles arrange at an interface as opposed to remaining dispersed in bulk? This study may be viewed as the starting point for subsequent investigations into the behaviour of coloured pigment particles at a range of fluid interfaces.
