# Supplementary material for: Pickering emulsions stabilized by coloured organic pigment particles
Source: Chem Sci. 2016 Sep 19;8(1):708–23. doi: 10.1039/c6sc03085h (PMC5465566; doi:10.1039/c6sc03085h)

Electronic Supplementary Material (ESI) for Chemical Science.
This journal is © The Royal Society of Chemistry 2016

ESI

Pickering Emulsions Stabilized by Coloured Organic Pigment Particles

Bernard P. Binks* and Samuel O. Olusanya

Department of Chemistry, University of Hull, Hull HU6 7RX, U.K.

Table S1. Elemental composition of the seven pure pigments.

Element	Composition/wt.%						
	PR	PO	PY	PG	PB	PI	PV
**C**	55.68	43.55	50.88	39.85	61.29	63.06	72.74
**O**	26.51	25.40	11.92	0.91	0.63	21.26	13.77
**N**	17.71	31.04	6.63	13.30	24.30	15.47	13.28
**S**	0.04	0.00	0.02	0.08	0.00	0.00	0.14
**Cl**	0.06	0.00	30.53	42.10	3.20	0.21	0.07
**Al**	0.00	0.01	0.02	0.25	0.00	0.00	0.00
**Cu**	0.00	0.00	0.00	3.51	10.58	0.00	0.00

Table S2. Values of the maximum wavelength and absorbance for each pigment (0.1 mg/ml) measured at room temperature in water and *n*-heptane for the unfiltered dispersion and its supernatant.

	***n*-Heptane**				**Water**			
Pigment	Dispersion		Supernatant		Dispersion		Supernatant	
	λ_{max}/nm	Abs.	λ_{max}/nm	Abs.	λ_{max}/nm	Abs.	λ_{max} /nm	Abs.
**PR**	600	0.133	351	0.106	607	0.292	364	0.094
**PO**	347	0.084	351	0.068	547	0.406	476	0.249
**PY**	490	0.022	352	0.148	479	0.108	465	0.062
**PG**	675	0.131	370	0.048	670	0.096	370	0.071
**PB**	647	0.424	410	0.047	384	0.177	370	0.096
**PI**	646	0.084	435	0.048	660	0.106	430	0.075
**PV**	610	0.107	610	0.039	610	0.098	590	0.060

Table S3. Values of apparent extinction coefficient for the seven pigments in water and *n*-heptane.

	***n*-Heptane**	**Water**
**Pigment**	ε/(mg/ml)$^{-1}$ cm^{-1}	ε/(mg/ml)$^{-1}$ cm^{-1}
**PR**	2.5	2.1
**PO**	0.3	28
**PY**	3.4	1.3
**PG**	6.7	2.4
**PB**	8.3	2.6
**PI**	1.2	0.8
**PV**	1.0	1.0

Figure S1. SEM images for the seven pigments at low magnification for (a) PR, (b) PO, (c) PY, (d) PG, (e) PB, (f) PI, (g) PV.

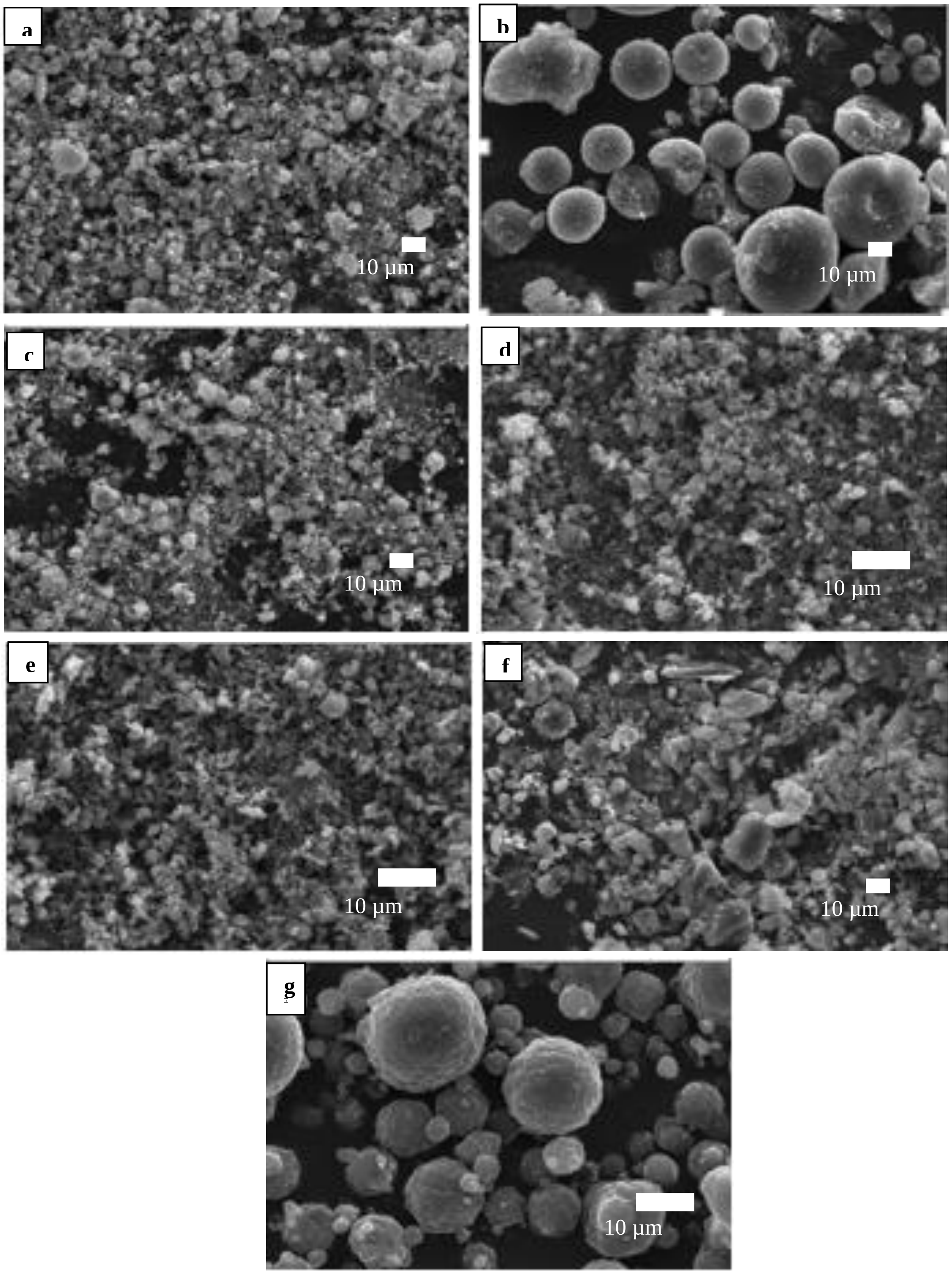

Figure S2. EDX spectra for the seven pigments for (a) PR, (b) PO, (c) PY, (d) PG, (e) PB, (f) PI, (g) PV.

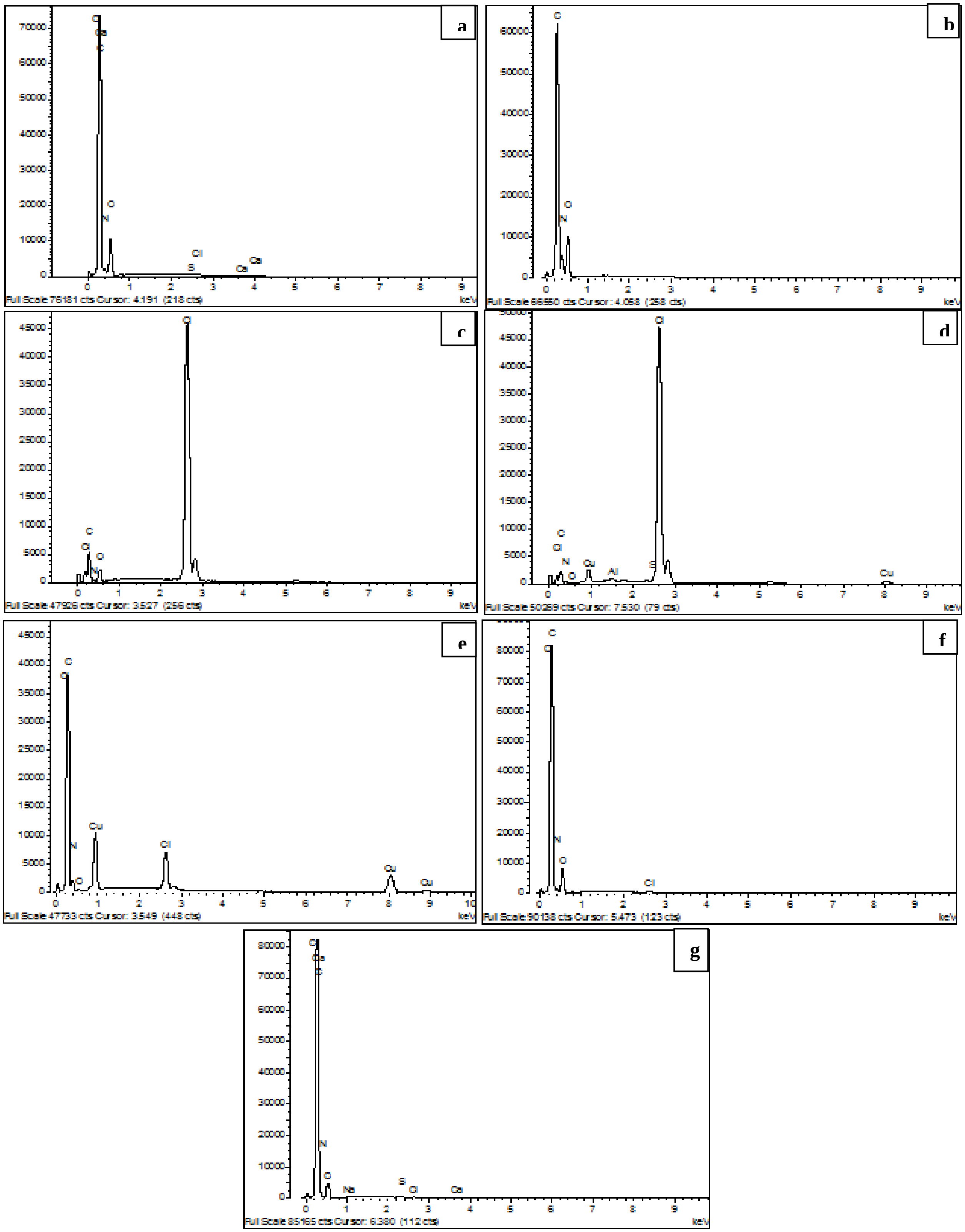

Figure S3. 3-D surface energy diagrams for pigment particles determined at various values of γ_{sa}^{d} and γ_{sa}^{p} for (a) PY, (b) PG, (c) PB, (d) PI.

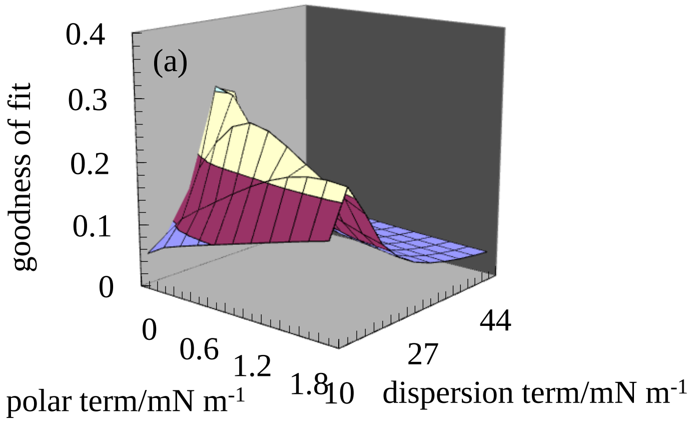

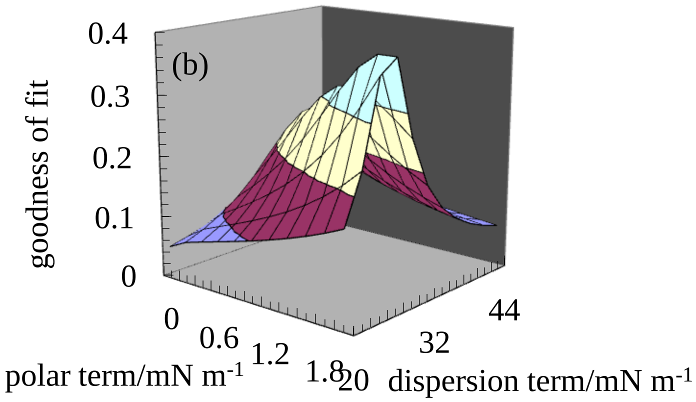

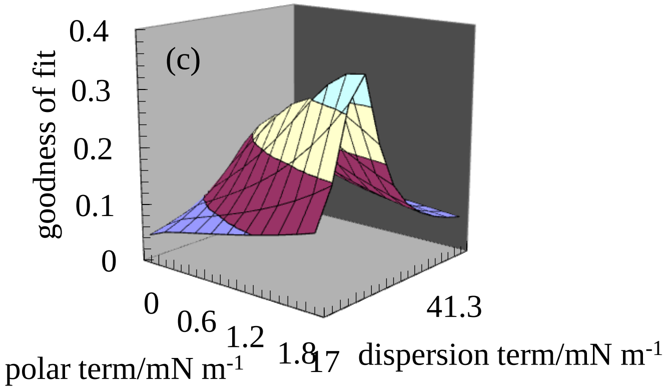

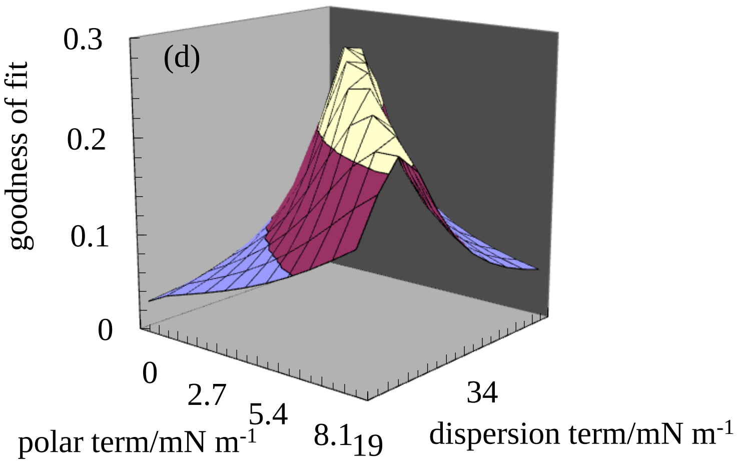

0.3
0.2
0.1
0
(d)
goodness of fit
0
2.7
5.4
8.1
19
34
polar term/mN m-1
dispersion term/mN m-1

Figure S4. Graphs of absorbance against wavelength determined with 0.1 mg/ml of each pigment dispersion and its supernatant in *n*-heptane (left column) and water (right column) for (i) PR, (ii) PO, (iii) PY, (iv) PG, (v) PB, (vi) PI and (vii) PV.

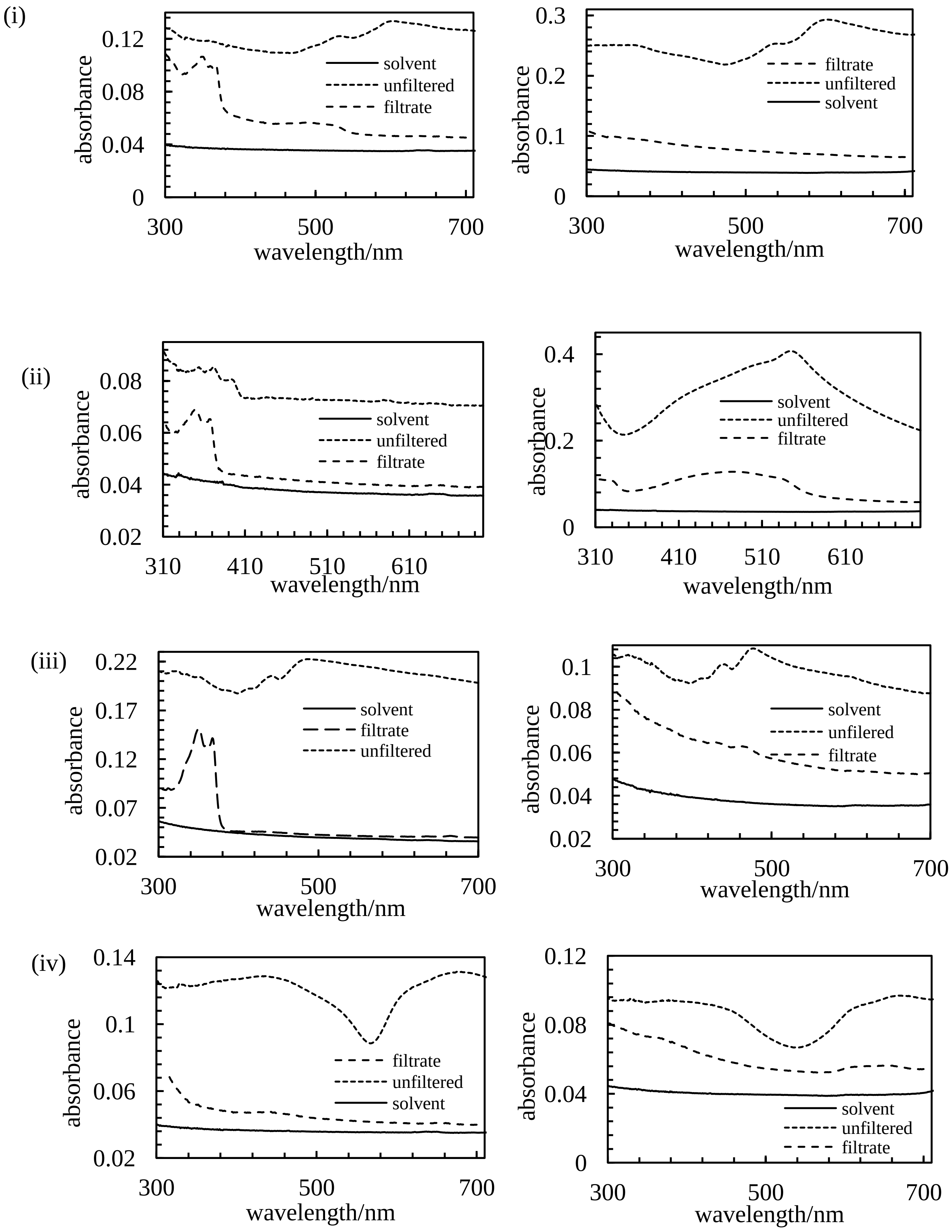

(v)

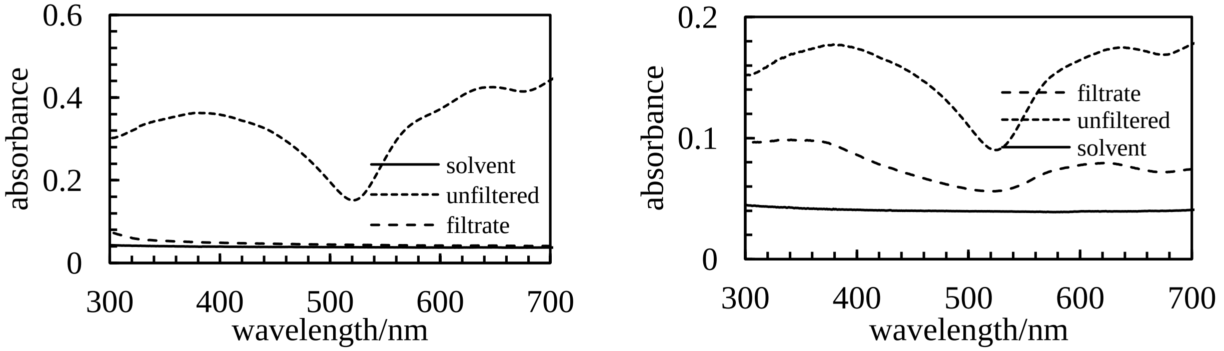
absorbance
wavelength/nm
solvent
unfiltered
filtrate
filtrate
unfiltered
solvent

(vi)

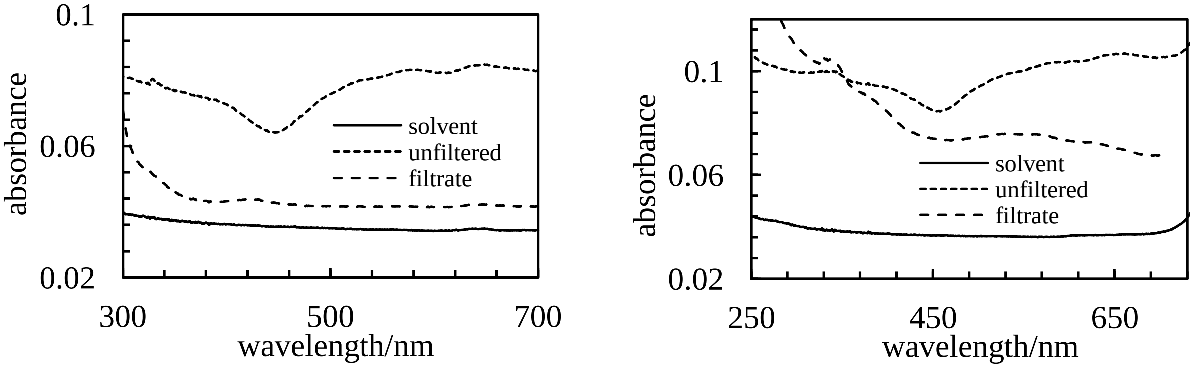
absorbance
wavelength/nm
solvent
unfiltered
filtrate
solvent
unfiltered
filtrate

(vii)

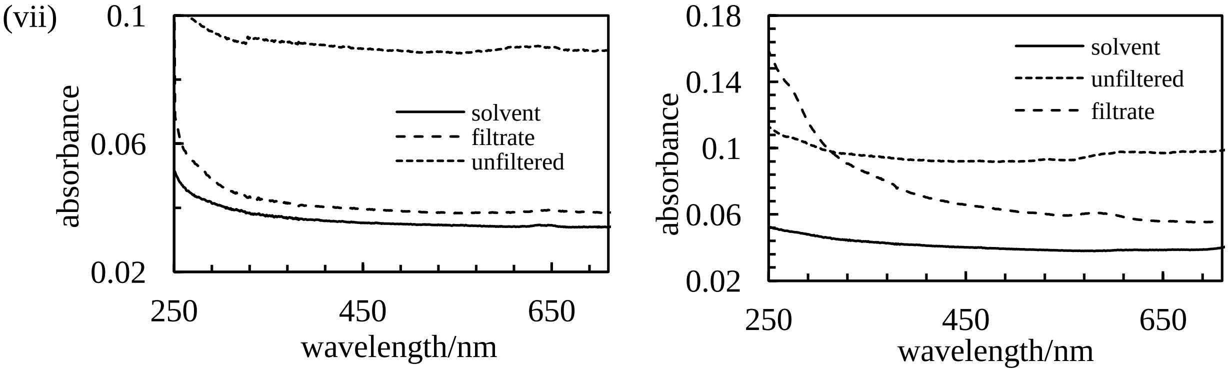
absorbance
wavelength/nm
solvent
filtrate
unfiltered
solvent
unfiltered
filtrate

Figure S5. Graph of absorbance against concentration for dispersions of the seven pigments in *n*-heptane (left column) or water (right column) for (i) PR, (ii) PO, (iii) PY, (iv) PG, (v) PB, (vi) PI and (vii) PV.

(i)

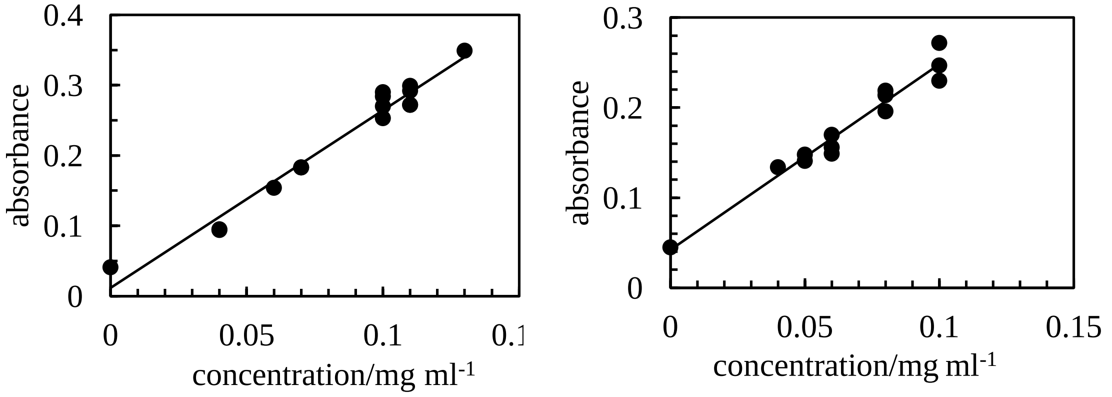

(ii)

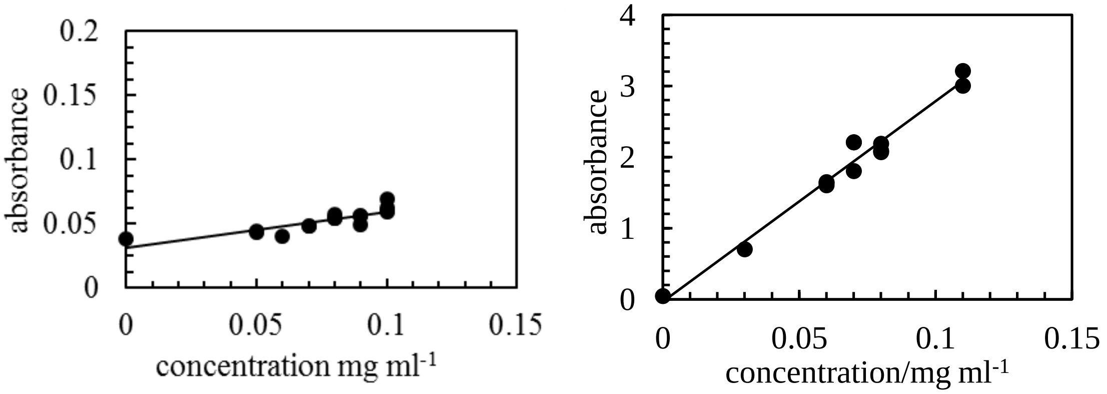

(iii)

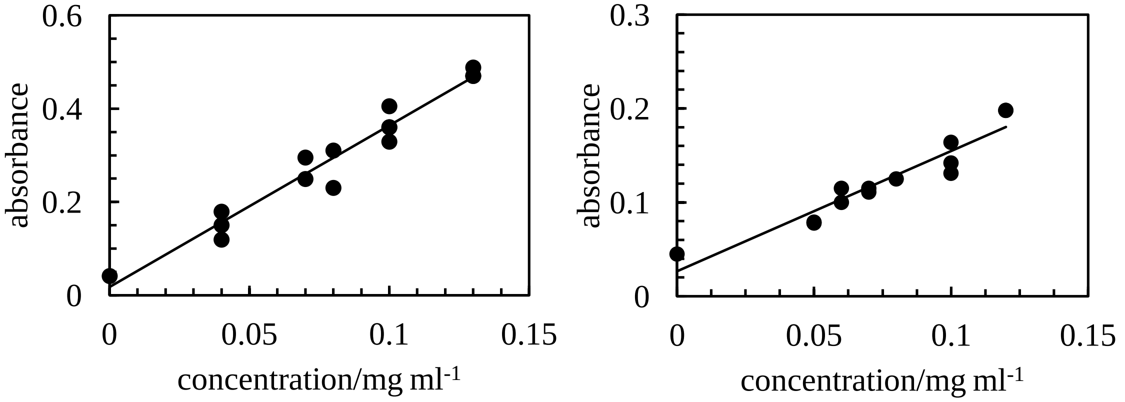

(iv)

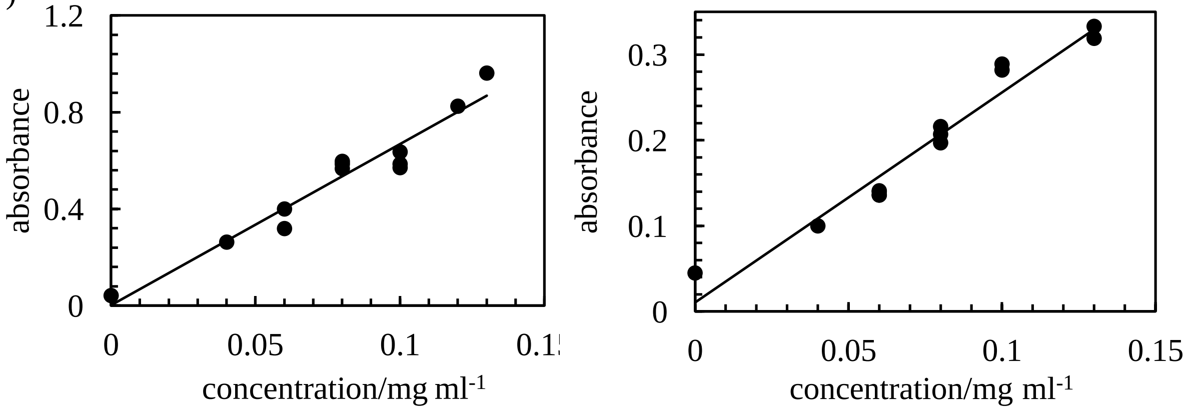

(v)

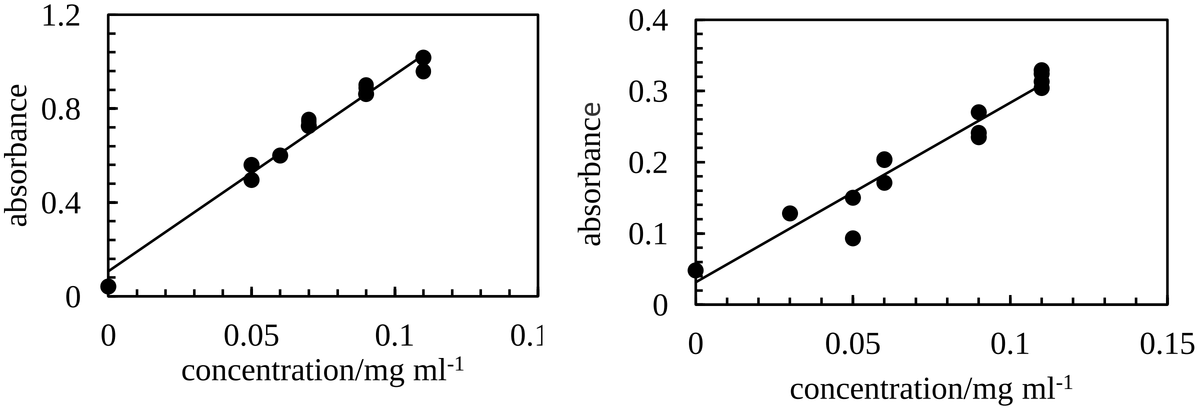
1.2
0.8
0.4
0
absorbance
0
0.05
0.1
0.1
concentration/mg ml-1
0.4
0.3
0.2
0.1
0
absorbance
0
0.05
0.1
0.15
concentration/mg ml-1

(vi)

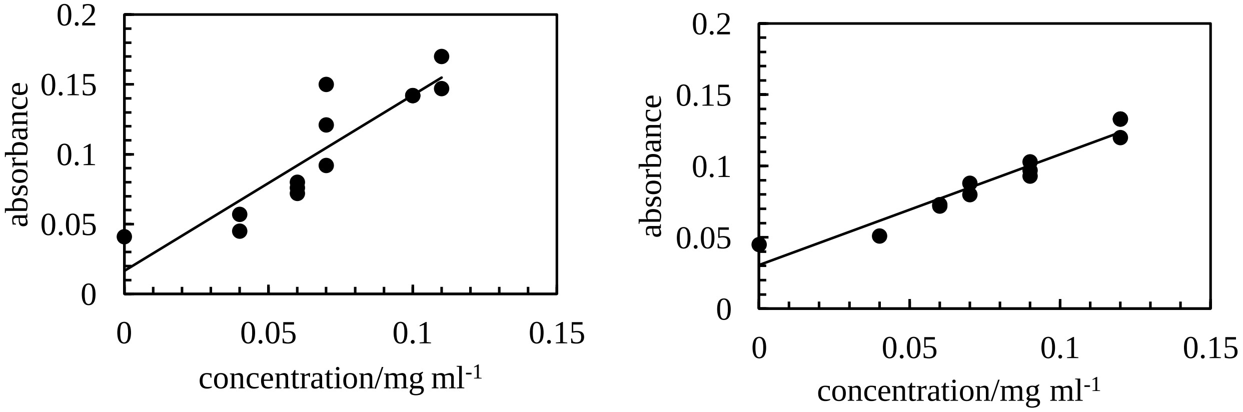
0.2
0.15
0.1
0.05
0
absorbance
0
0.05
0.1
0.15
concentration/mg ml-1
0.2
0.15
0.1
0.05
0
absorbance
0
0.05
0.1
0.15
concentration/mg ml-1

(vii)

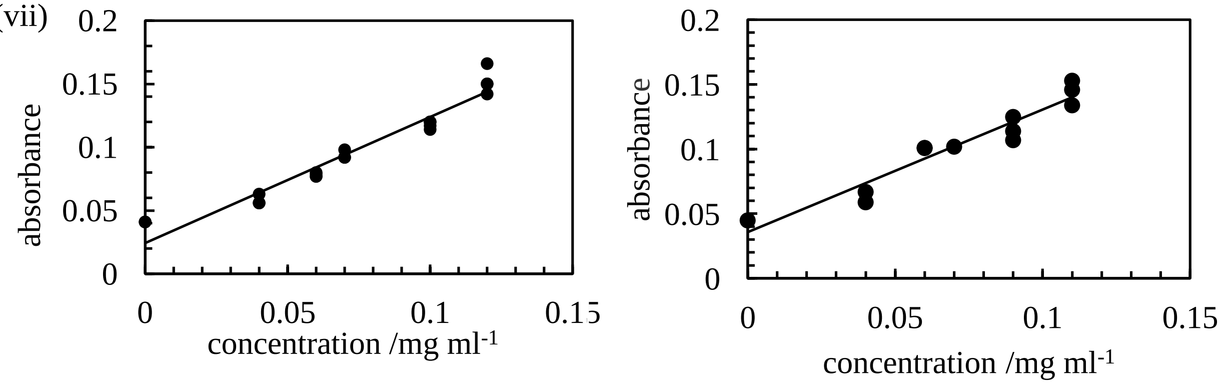
0.2
0.15
0.1
0.05
0
absorbance
0
0.05
0.1
0.15
concentration /mg ml-1
0.2
0.15
0.1
0.05
0
absorbance
0
0.05
0.1
0.15
concentration /mg ml-1

Figure S6. Fraction of (a) oil and (b) water resolved as a function of time for *n*-heptane-water emulsions stabilized by 1 wt.% of the different pigments at $\phi_w = 0.5$.

(a)

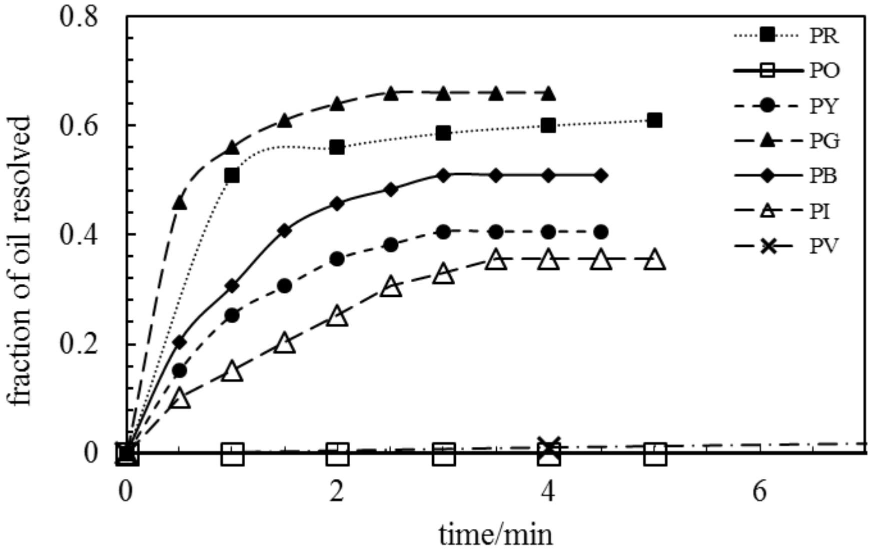

(b)

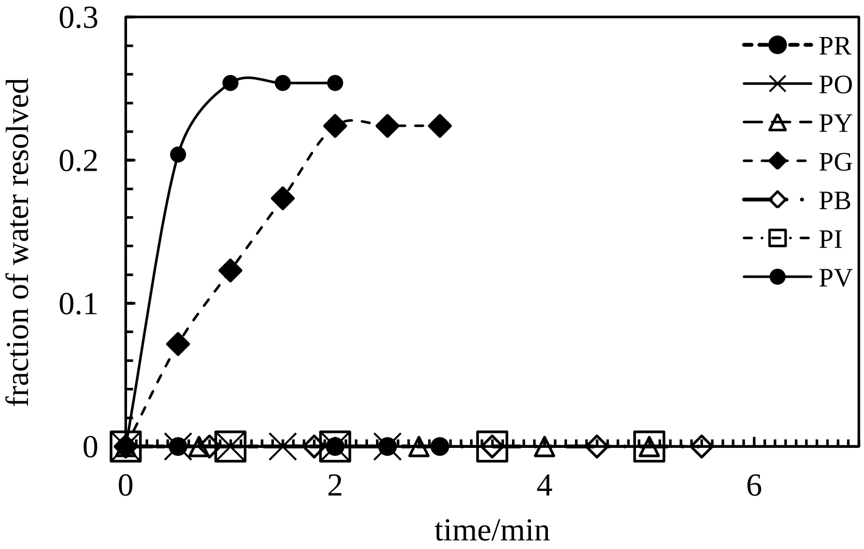

Figure S7. Optical micrographs of fresh emulsions $\phi_w = 0.5$ stabilized by the seven pigments at different particle concentrations for (a) PR, (b) PO, (c) PY, (d) PG, (e) PB, (f) PI, (g) PV.

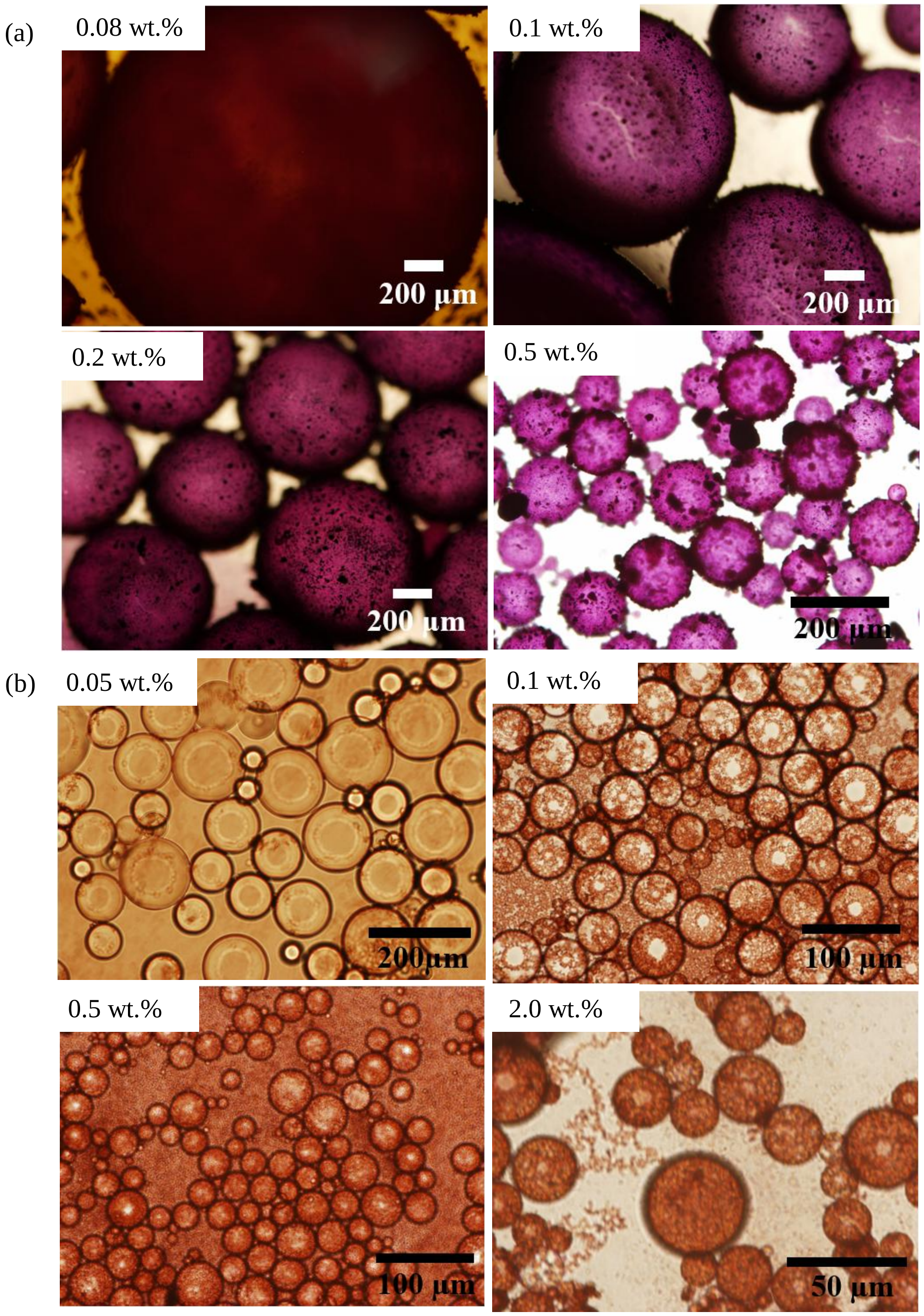

(c)

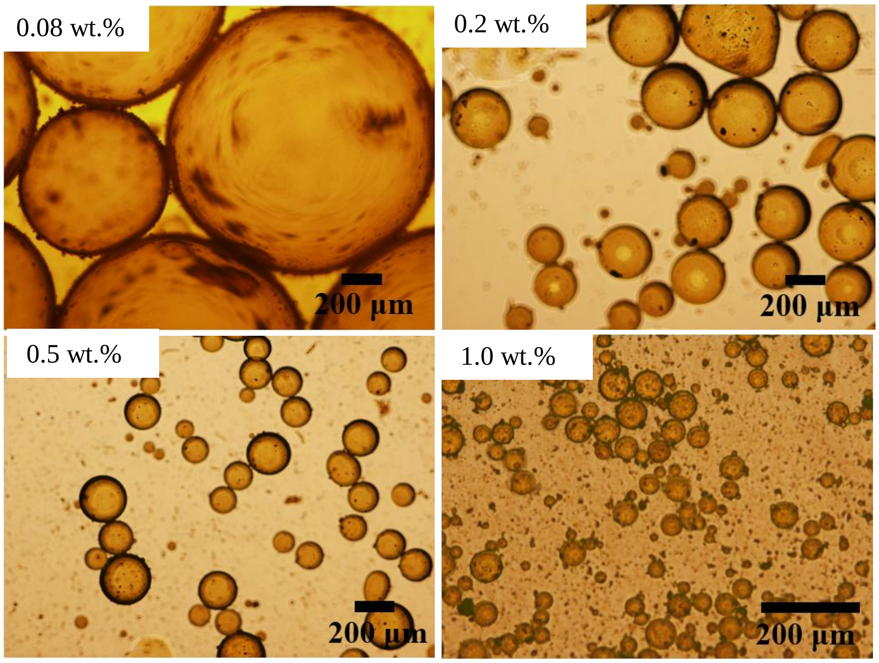
0.08 wt.%
200 µm
0.2 wt.%
200 µm
0.5 wt.%
200 µm
1.0 wt.%
200 µm

(d)

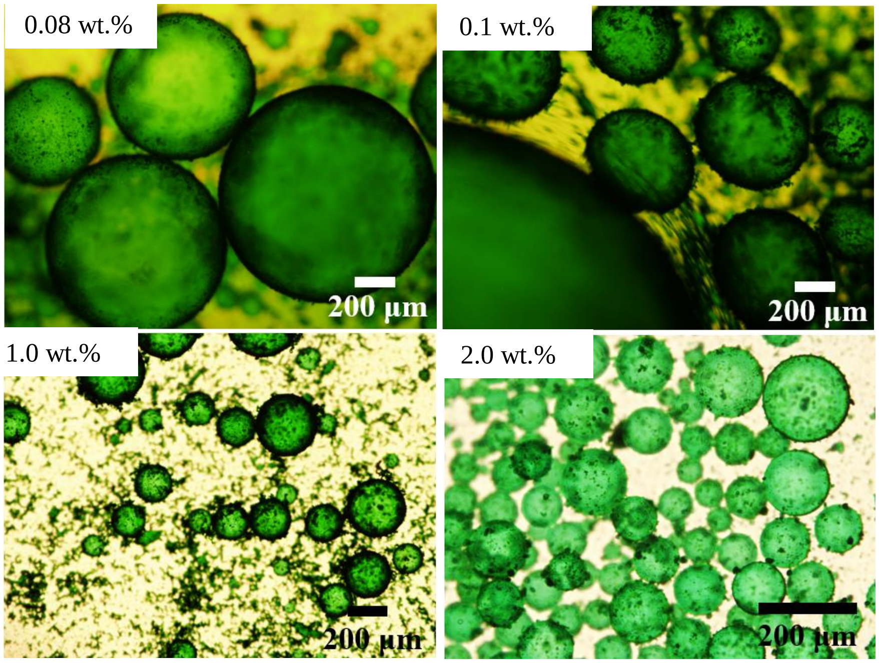
0.08 wt.%
200 µm
0.1 wt.%
200 µm
1.0 wt.%
200 µm
2.0 wt.%
200 µm

(e)

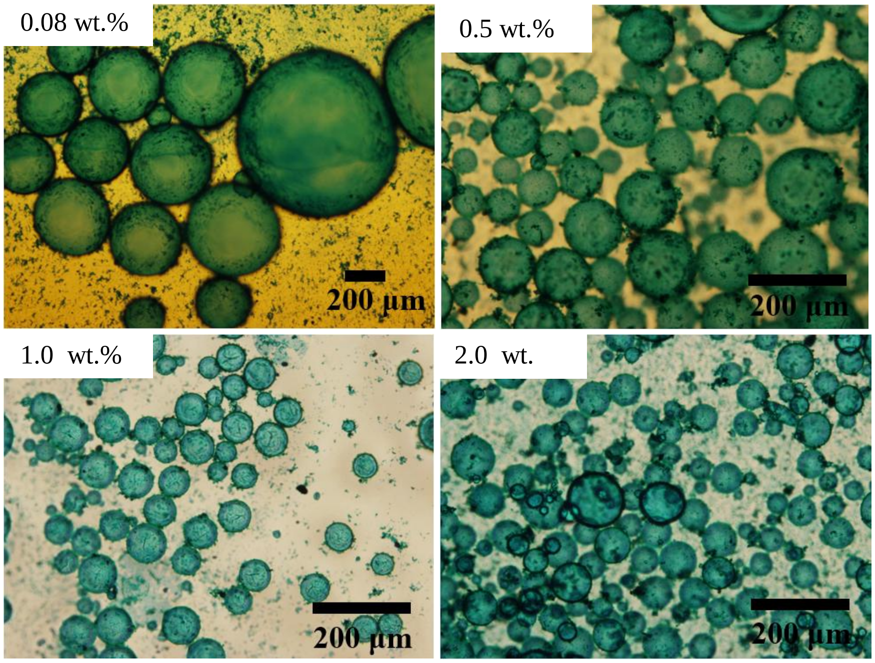
0.08 wt.%
200 μm
0.5 wt.%
200 μm
1.0 wt.%
200 μm
2.0 wt.
200 μm

(f)

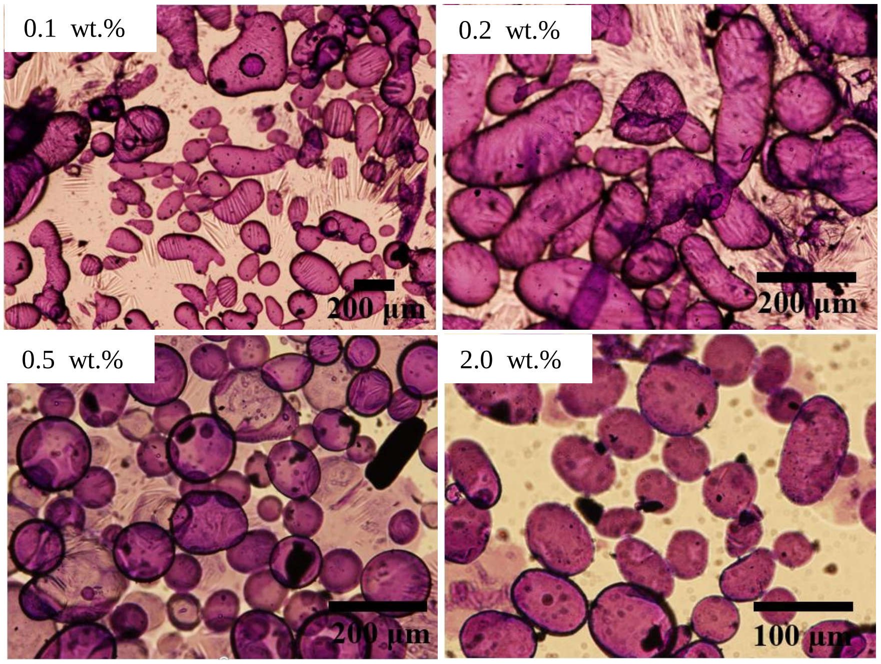
0.1 wt.%
200 μm
0.2 wt.%
200 μm
0.5 wt.%
200 μm
2.0 wt.%
100 μm

(g)

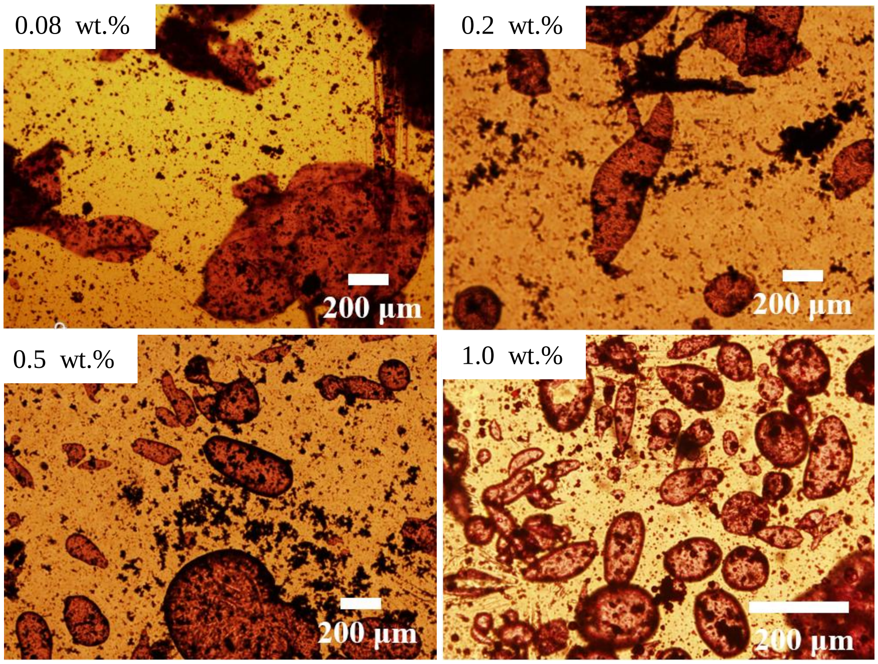

Figure S8. Variation of emulsion conductivity as a function of water volume fraction for 1 wt.% pigment-stabilized emulsions for (a) PR, (b) PY, (c) PB, (d) PI. Vertical dotted line signifies phase inversion.

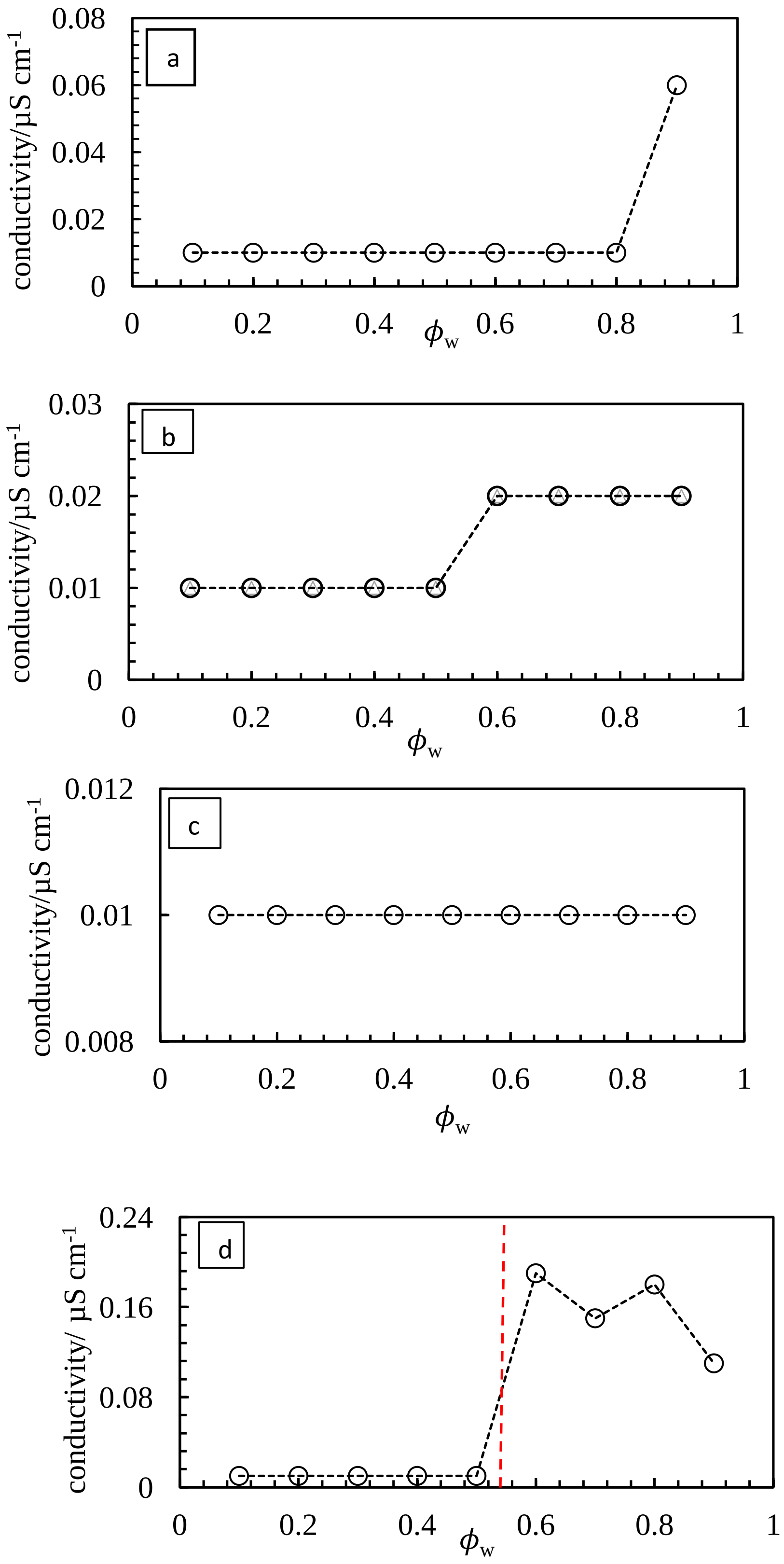

Figure S9. Variation of fraction of oil (filled points) and water (open points) resolved as a function of initial water volume fraction two months after preparation for emulsions stabilized by (a) PR, (b) PY, (c) PB, (d) PI.

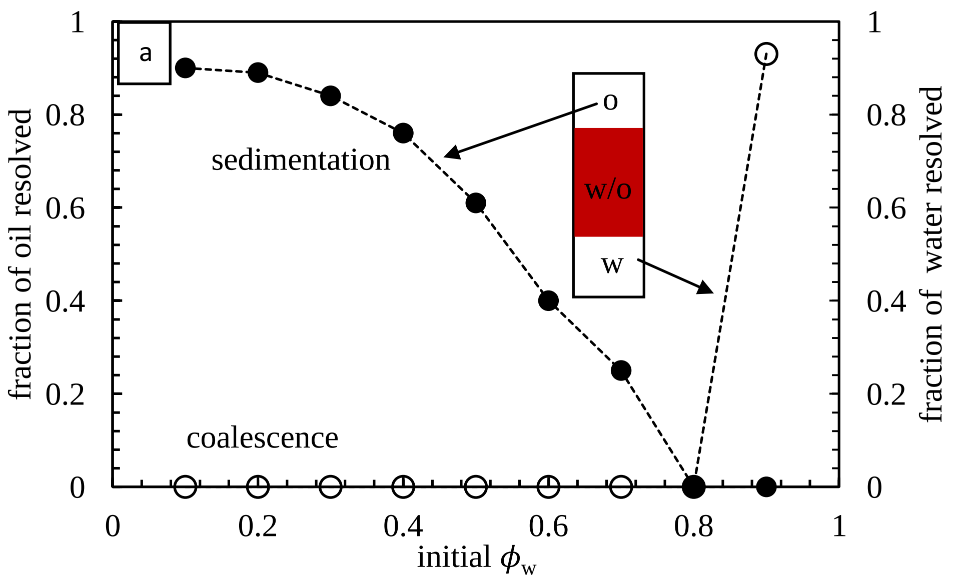

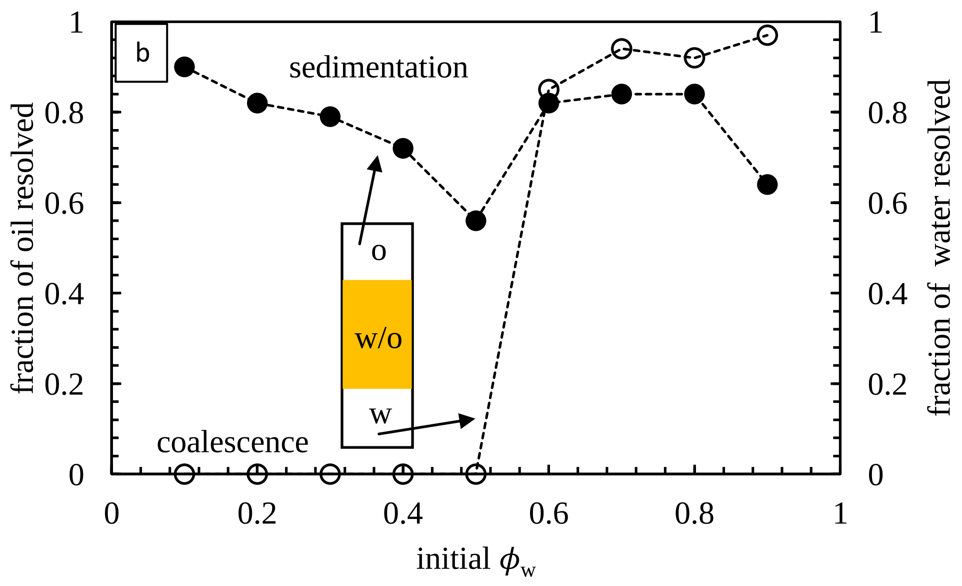

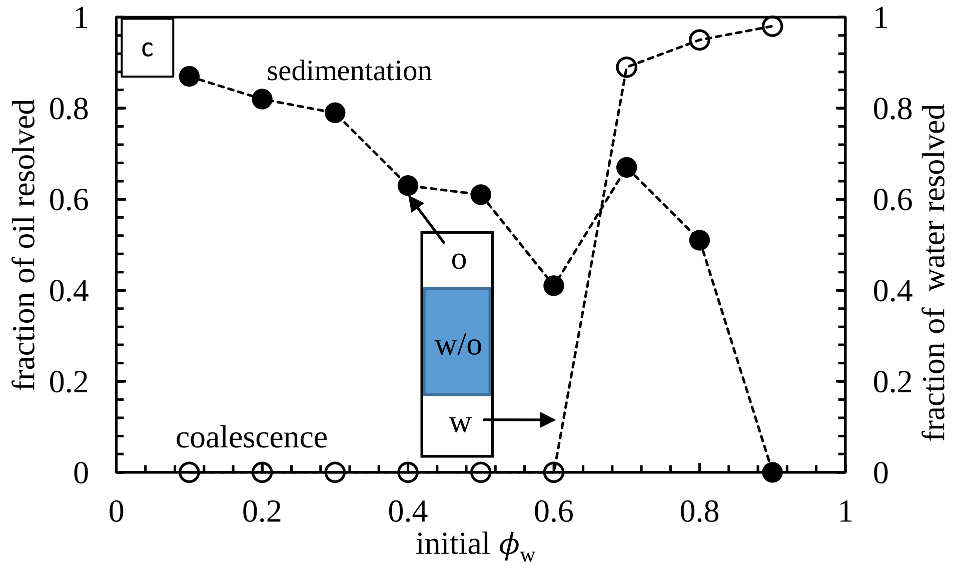

c
sedimentation
coalescence
o
w/o
w
fraction of oil resolved
fraction of water resolved
initial ϕ_w

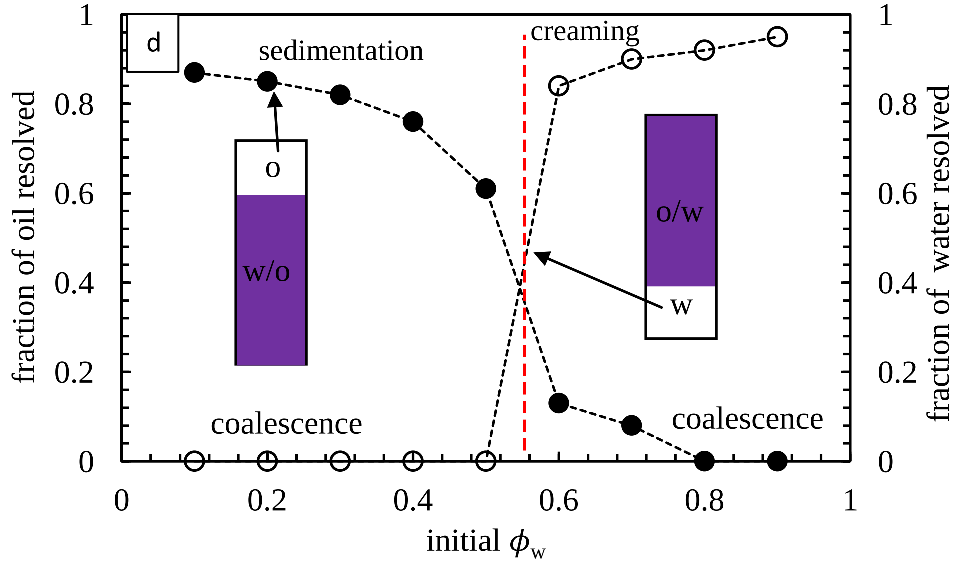

d
sedimentation
creaming
o
w/o
o/w
w
coalescence
coalescence
fraction of oil resolved
fraction of water resolved
initial ϕ_w

Figure S10. Optical micrographs of emulsions of Figure S8 after 20 min at different water volume fractions (given) for (a) PR, (b) PO, (c) PY, (d) PG, (e) PB, (f) PI, (g) PV.

(a)

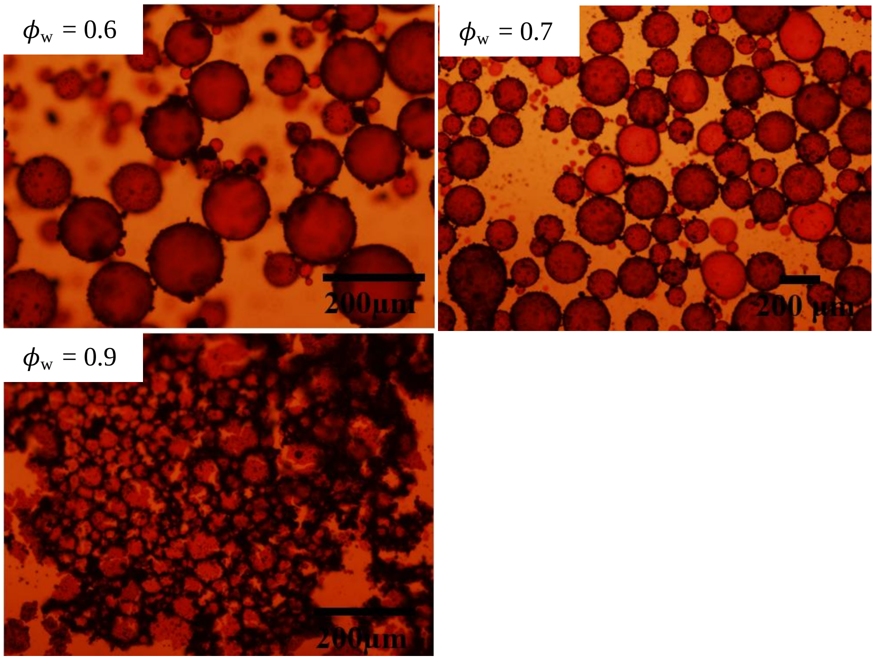

(b)

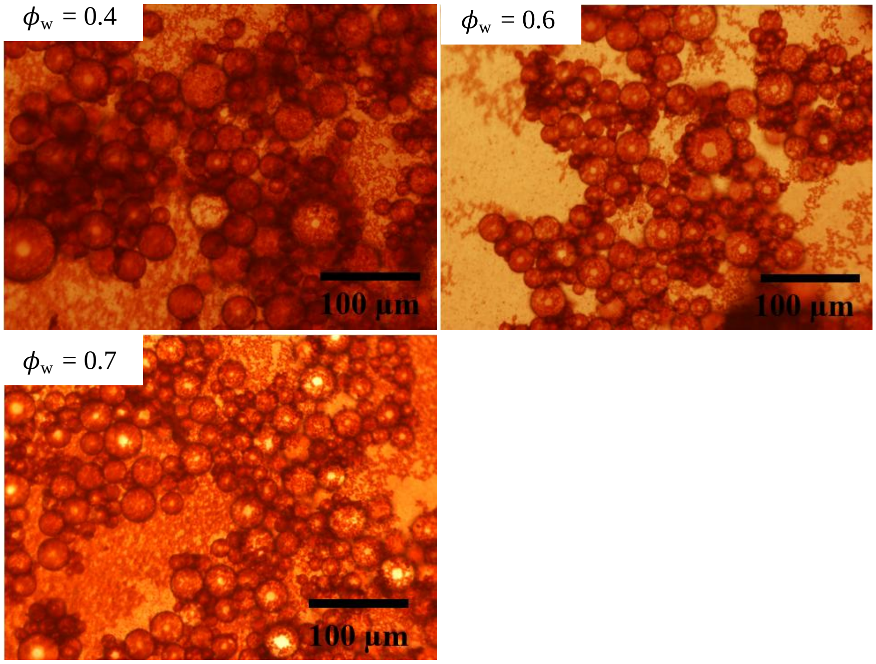

(c)

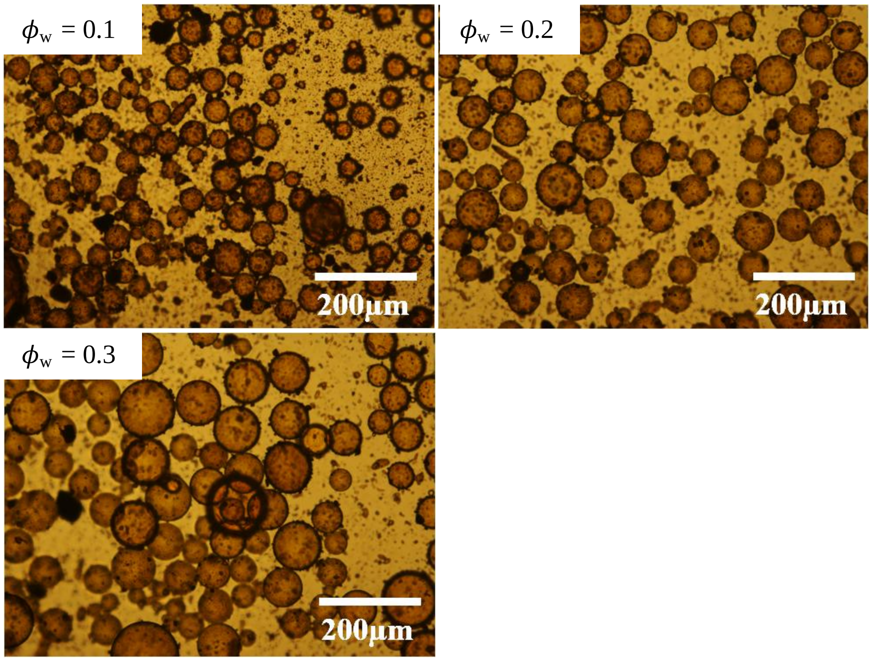

ϕ_w = 0.1
200µm
ϕ_w = 0.2
200µm
ϕ_w = 0.3
200µm

(d)

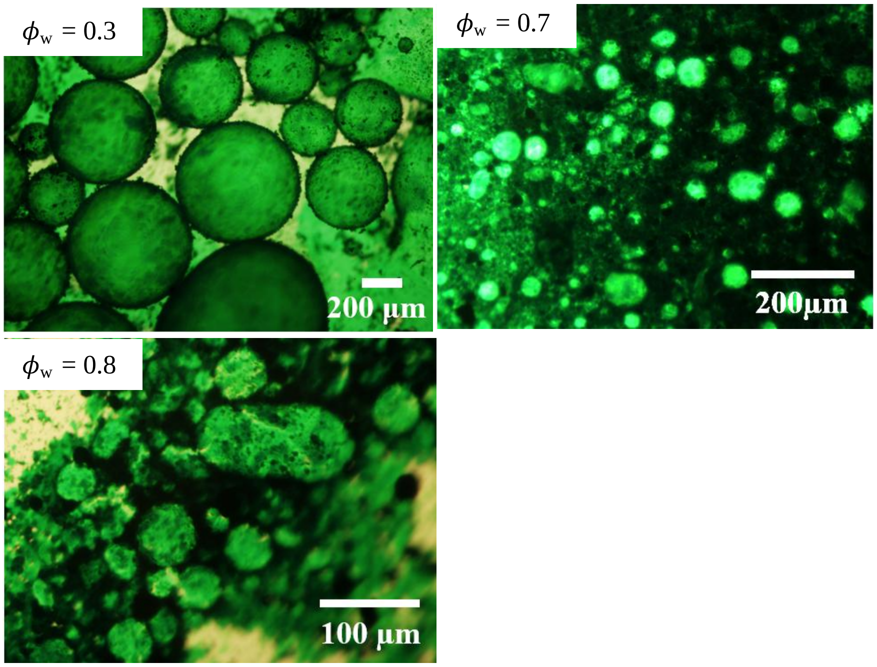

ϕ_w = 0.3
200 µm
ϕ_w = 0.7
200µm
ϕ_w = 0.8
100 µm

(e)

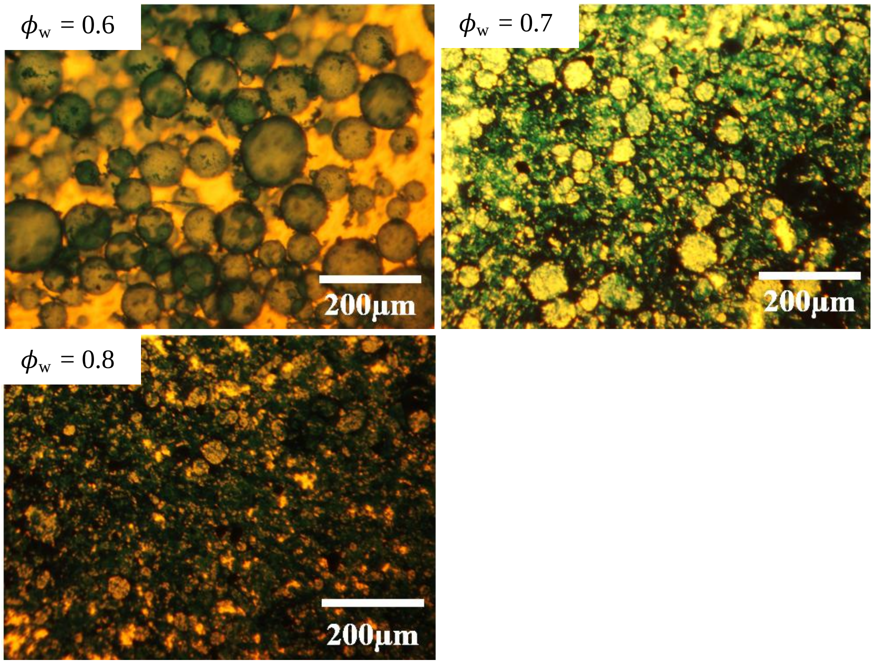
ϕ_w = 0.6
200µm
ϕ_w = 0.7
200µm
ϕ_w = 0.8
200µm

(f)

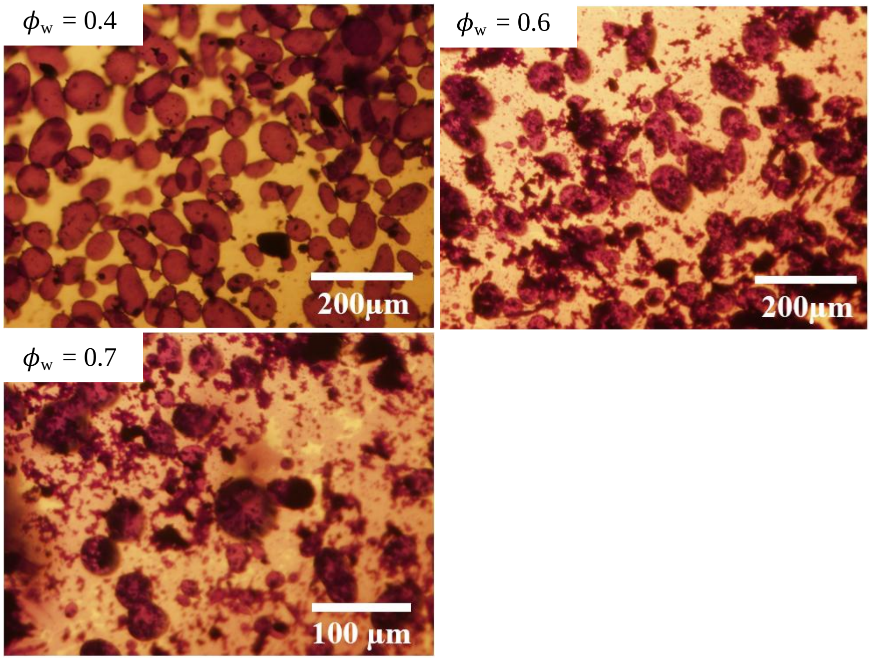
ϕ_w = 0.4
200µm
ϕ_w = 0.6
200µm
ϕ_w = 0.7
100 µm

(g)

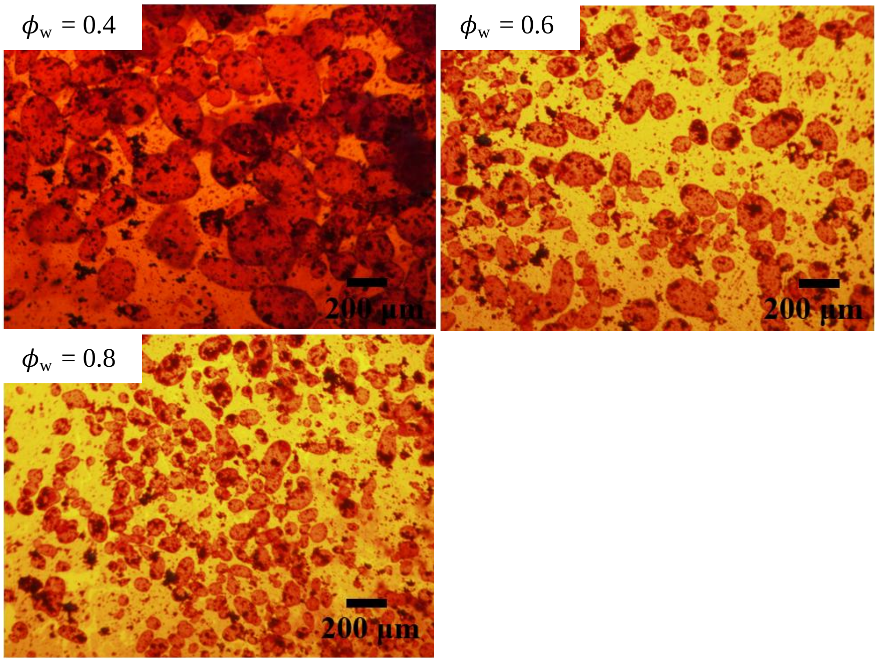

Figure S11. Variation of drop diameter with aqueous volume fraction for emulsions stabilised by 1 wt.% of pigment for (a) PR, (b) PY (c) PB, (d) PI.

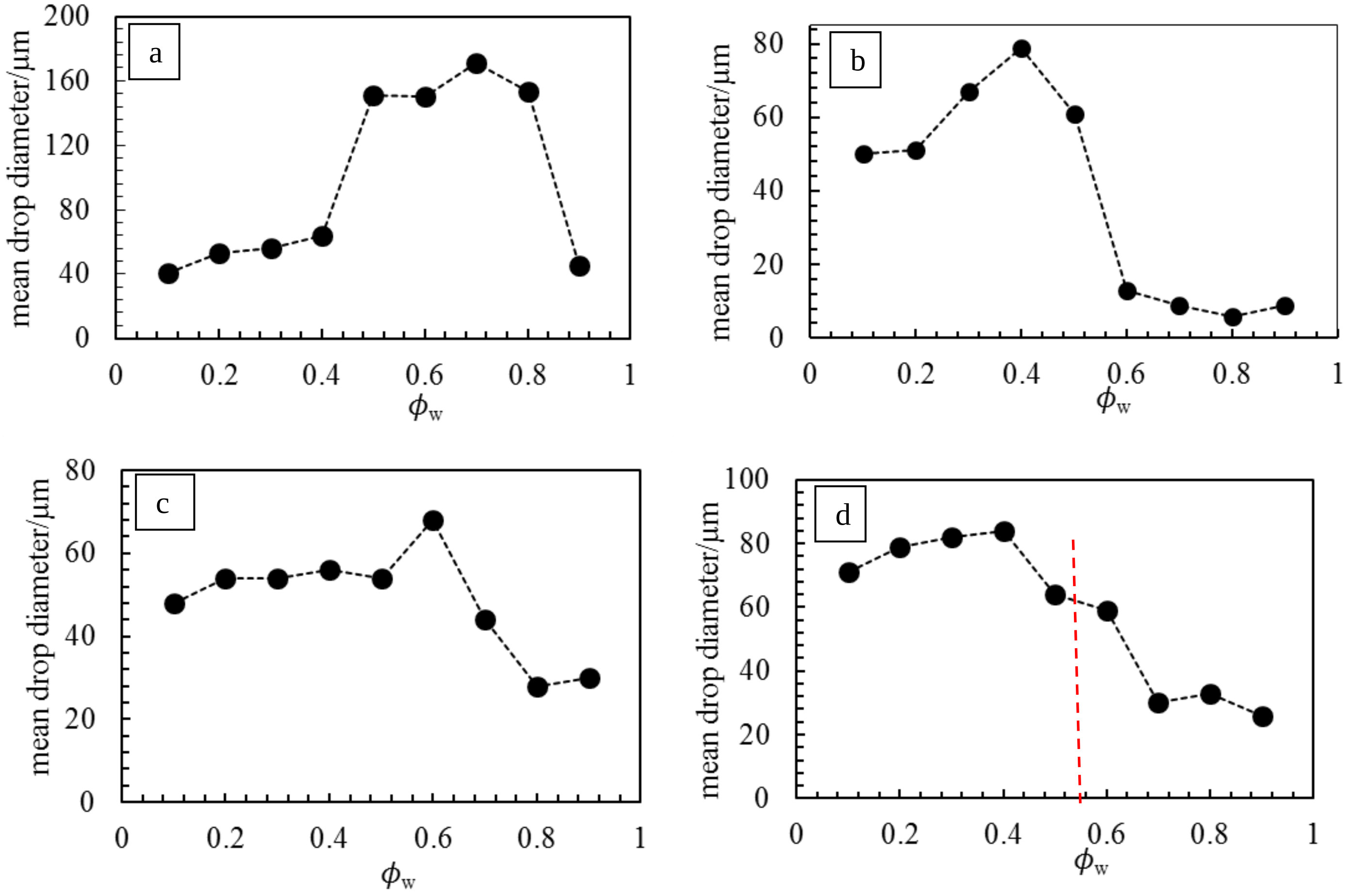

Supplement: Supplementary file 1 [file SC-008-C6SC03085H-s001.pdf]
